# Role of signaling pathways in age-related orthopedic diseases: focus on the fibroblast growth factor family

**DOI:** 10.1186/s40779-024-00544-5

**Published:** 2024-06-21

**Authors:** Heng-Zhen Li, Jing-lve Zhang, Dong-Liang Yuan, Wen-Qing Xie, Christoph H. Ladel, Ali Mobasheri, Yu-Sheng Li

**Affiliations:** 1 Department of Orthopedics, Xiangya Hospital, Central South University, Changsha, 410008 China; 2 National Clinical Research Center for Geriatric Disorders, Xiangya Hospital, Central South University, Changsha, 410008 China; 3 Department of Plastic and Cosmetic Surgery, Xiangya Hospital, Central South University, Changsha, 410008 China; 4 Xiangya School of Medicine Central, South University, Changsha, 410083 China; 5 CHL4special consulting, 64291 Darmstadt, Germany; 6 Faculty of Medicine, Research Unit of Health Sciences and Technology, University of Oulu, 90014 Oulu, Finland; 7 Department of Regenerative Medicine, State Research Institute Centre for Innovative Medicine, 08406 Vilnius, Lithuania; 8 Department of Rheumatology and Clinical Immunology, Universitair Medisch Centrum Utrecht, Utrecht, 3508 GA the Netherlands; 9 Department of Joint Surgery, the First Affiliated Hospital of Sun Yat-sen University, Guangzhou, 510080 China; 10 World Health Organization Collaborating Centre for Public Health Aspects of Musculoskeletal Health and Aging, Université de Liège, B-4000 Liège, Belgium

**Keywords:** Fibroblast growth factor (FGF), Fibroblast growth factor receptor (FGFR), Osteoarthritis (OA), Intervertebral disc degeneration (IVDD), Orthopedic degeneration, Osteoporosis (OP), Sarcopenia

## Abstract

Fibroblast growth factor (FGF) signaling encompasses a multitude of functions, including regulation of cell proliferation, differentiation, morphogenesis, and patterning. FGFs and their receptors (FGFR) are crucial for adult tissue repair processes. Aberrant FGF signal transduction is associated with various pathological conditions such as cartilage damage, bone loss, muscle reduction, and other core pathological changes observed in orthopedic degenerative diseases like osteoarthritis (OA), intervertebral disc degeneration (IVDD), osteoporosis (OP), and sarcopenia. In OA and IVDD pathologies specifically, FGF1, FGF2, FGF8, FGF9, FGF18, FGF21, and FGF23 regulate the synthesis, catabolism, and ossification of cartilage tissue. Additionally, the dysregulation of FGFR expression (FGFR1 and FGFR3) promotes the pathological process of cartilage degradation. In OP and sarcopenia, endocrine-derived FGFs (FGF19, FGF21, and FGF23) modulate bone mineral synthesis and decomposition as well as muscle tissues. FGF2 and other FGFs also exert regulatory roles. A growing body of research has focused on understanding the implications of FGF signaling in orthopedic degeneration. Moreover, an increasing number of potential targets within the FGF signaling have been identified, such as FGF9, FGF18, and FGF23. However, it should be noted that most of these discoveries are still in the experimental stage, and further studies are needed before clinical application can be considered. Presently, this review aims to document the association between the FGF signaling pathway and the development and progression of orthopedic diseases. Besides, current therapeutic strategies targeting the FGF signaling pathway to prevent and treat orthopedic degeneration will be evaluated.

## Background

In mammals, the fibroblast growth factor (FGF) family consists of 18 highly conserved secreted signaling proteins capable of binding and activating 4 receptor tyrosine kinases, thereby regulating downstream signaling pathways [1]. Fifteen classical FGF signaling molecules are known to be important in the early stages of embryonic growth and organ formation. They are persistently expressed in adult tissues as they are pivotal for regulating tissue growth, regeneration, repair, and metabolic homeostasis [2]. FGF15, FGF19, and FGF21 exhibit expression across multiple tissues and exert essential functions during the initial stages of embryonic development. Anormal in FGF signaling pathways has been associated with various diseases, including osteoarthritis (OA), cancer, heart disease, angiogenesis disorders, achondroplasia, and impaired embryonic development

Orthopedic degenerative diseases, including OA, intervertebral disc degeneration (IVDD), osteoporosis (OP), and sarcopenia, are prevalent among the elderly population. With the increasing aging population, there is a rise in the incidence of these diseases, making the progression of pathological degeneration a significant public health concern. These disease conditions lead to reduced mobility, and increased muscle and joint pain, greatly impacting the quality of life for older individuals. This poses a substantial burden on both global public health and rehabilitation healthcare systems. Orthopedic degenerative diseases involve various pathological changes, such as long-term chronic inflammation, joint cartilage degradation, and abnormal subchondral bone formation. These processes are often regulated by numerous signaling pathways in the body. Signaling pathways like transforming growth factor-β, Wnt/β-catenin, and hypoxia-inducible factor play important roles in regulating cartilage growth and development, bone mineral density (BMD), and muscle mass associated with orthopedic degenerative diseases [3–6]. The FGF signaling pathway has gained attention from medical researchers due to its involvement in skeletal muscle growth and development regulation as well as extracellular matrix (ECM) synthesis and decomposition, and bone tissue signal transduction [7, 8]. Various members of the FGF family contribute differently to chronic pathological changes seen in orthopedic degenerative diseases. For example, FGF1 and FGF8 may contribute to cartilage destruction during the development of OA [9, 10], whereas FGF18 can promote cartilage repair and protect joint cartilage integrity. Additionally, FGF18 inhibits apoptosis of nucleus pulposus (NP) cells protecting intervertebral discs (IVDs) affected by IVDD [11]. In cases of sarcopenia and OP, FGF shows potential for slowing down or reversing disease progression. The therapeutic applications targeting the FGF family have garnered significant attention, with ongoing clinical trials focused on FGF18. Therefore, this review emphasizes the role of the FGF signaling pathway in the pathogenesis and progression of orthopedic degenerative diseases and assesses its potential as a molecular target for the prevention and treatment of these conditions.

## FGFs and fibroblast growth factor receptors (FGFRs)

### Structure of FGFs and FGFRs

The FGF superfamily has 22 members, with 4 of them being intracellular proteins that do not bind to extracellular receptors. The remaining 18 members (FGF1-10 and FGF16-23) act as ligands, binding to receptor tyrosine kinases. FGFs share a similar core structure comprising 120 – 130 amino acids arranged in 12 antiparallel β chains (β1-12), which exhibit high affinity for FGFRs and heparin [12]. The mammalian FGFR family is encoded by 4 FGFR genes (FGFR1-4), consisting of 3 extracellular immunoglobulin domains (D1-D3), a cytoplasmic tyrosine kinase domain, and a one-way transmembrane domain. These receptors possess highly conserved sequences but differ primarily in terms of ligand affinity and tissue distribution [13, 14]. An acidic sequence rich in serine known as the acid box is a distinctive feature located between D1 and D2 in the extracellular immunoglobulin domain of FGFRs [15]. During receptor function, the acid box and D1 domain play roles in receptor self-inhibition, whereas the D2 and D3 domains are involved in ligand-specific binding. The transmembrane domain anchors the receptor to the cell membrane facilitating receptor dimerization. Additionally, the near-membrane region of the FGFR also contributes to receptor dimerization [16]. Most FGFs act as paracrine factors during organogenesis. FGF19, FGF21, and FGF23 regulate the homeostasis of phosphate, vitamin D, cholesterol, and glucose, among other substances in the endocrine system [2, 17].

### FGF-FGFR interaction

The binding of FGF ligands to FGFR initiates the FGF signaling cascade in a heparan sulfate glycosaminoglycan-dependent manner, facilitating FGF-FGFR dimerization. This process promotes and stabilizes the 1:1 ratio between the ligand and receptor within the FGF-FGFR complex, as well as the 2:2 protein-protein contact in the FGF-FGFR dimer. Moreover, it contributes to stabilizing FGF against degradation. This complex consists of two FGFs, two heparin sulfate chains, and two FGFRs [18, 19]. Upon FGFR dimerization, their cytoplasmic kinase domains undergo transphosphorylation at cyclic tyrosine residues, leading to activation. It has been determined that FGFR1 has at least 7 phosphorylation sites (Tyr163, Tyr583, Tyr585, Tyr653, Tyr654, Tyr730, and Tyr766) [20, 21]. Following cyclic phosphorylation, tyrosine phosphorylation occurs at the C-terminus end near both the insertion and membrane regions of the kinase domain. The phosphotyrosine group serves as a site for downstream signal-binding proteins.

The binding of different FGFs and FGFRs elicits various in vivo effects. For instance, when FGF1 binds to FGFR1-3 IgIIIc subtypes, it quickly directs the receptor to the lysosome, whereas FGFR4 is directed toward the recycling region [22]. There are two speculative explanations for this phenomenon. Firstly, distinct intracellular signaling pathways are activated by different combinations of FGF-FGFR. Secondly, the function of FGF-FGFR can be influenced by the target cell type and its surrounding environment. For example, one type of cell responds to FGFs by activating a specific intracellular pathway, whereas another type utilizes an entirely different mechanism. Although both speculative mechanisms may determine the function of FGF-FGFR in vivo, most studies have provided evidence supporting the second mechanism involving the specific nature of the target cell and its microenvironment [23, 24]. Furthermore, when chimeric receptors were formed by linking different types of FGFR cytoplasmic domains with the extracellular domain of platelet-derived growth factor (PDGF) receptor, it was observed that this affected the intensity of tyrosine kinase activity rather than altering the identity of the target protein [25]. Therefore, quantitative or qualitative changes can also arise from variations in activation intensity within the FGF-FGFR system.

### Common downstream signaling of FGF-FGFR

The two intracellular substrates of FGFRs are phospholipase C (PLC)-γ and FGFR substrates 1 and 2 (FRS1 and FRS2). FGFR-invariant tyrosine residue at the C-terminus, specifically y766 in FGFR1, undergoes phosphorylation to form the SH2 structural domain binding site in PLC-γ. This phosphorylation event is necessary for both PLC-γ activation and its subsequent phosphorylation. FRS2 binds to the near-membrane region of FGFR and is phosphorylated by the receptor, thereby activating the Ras-mitogen-activated protein kinase (MAPK) and phosphatidylinositol-3-kinase (PI3K)-protein kinase B (Akt) signaling pathways. Consequently, these pathways including PLC-γ, RAS-MAPK, PI3K-Akt, as well as signal transduction and activator of transcription (STAT) pathways represent the most prevalent signaling pathways in the FGF signaling system [26–30].

#### RAS-MAPK pathway

MAPKs are a group of serine-threonine protein kinases that are activated by various extracellular stimuli and regulate cellular processes both in vivo and in vitro. The effectors of MAPK include c-Jun N-terminal kinase (JNK), extracellular signal-regulated kinase (ERK), and p38 mitogen-activated kinase [31]. The RAS-MAPK signaling pathway serves as the principal downstream pathway of FGF signaling, controlling cell proliferation and differentiation [32]. Upon binding to the receptor, FGF activates the phosphorylation of FRS2α, leading to the formation of the FRS2 complex that consists of FRS2α, guanine nucleotide exchange factor 2 (GRB2), GRB2-associated binding protein 1 (GAB1), son of sevenless (SOS), and tyrosine phosphatases. This complex activation subsequently triggers RAS and MAPK activation, ultimately resulting in ERK1/2 phosphorylation [33]. FGF also influences p38 phosphorylation and induces JNK-dependent proteasomal degradation of insulin receptor substrate 1 [34, 35]. Previous studies have demonstrated that the MAPK signaling pathway can reciprocally affect FGF function [36, 37]; for example, ERK1 and ERK2 directly phosphorylate FGFR1 on specific serine residues within its C-terminal region which significantly reduces tyrosine phosphorylation of the receptor kinase domain along with its associated with signalling [37]. Similarly, active ERKs also target multiple threonine residues in the docking protein FRS2, a key mediator involved in FGFR signaling [37]. Therefore, we propose further investigations should delve deeper into understanding the intricate interactions between these two pathways.

#### PI3K-Akt pathway

The findings demonstrate that FGF can recruit GAB1 to activate PI3K and the anti-apoptotic protein kinase Akt through the previously mentioned FRS2α [27]. Moreover, the anti-apoptotic and autophagy-regulating effects of FGF are closely associated with the PI3K-Akt signaling pathway. For example, an increase in autophagy induced by FGF-18 occurs after stimulation of Akt-transient receptor potential mucolipid 1-calponemalignin signaling pathway [38], while FGF2 and FGF4 promote the proliferation of stem cell antigen-1 (Sca-1)+ bone marrow mesenchymal stem cells through the activation of ERK1/2 and PI3K-Akt signaling pathways [39].

#### PLC-γ

After FGFR kinase activation, PLC-γ is recruited and hydrolyzed by phosphatidylinositol(4,5)bisphosphate (PIP2) to generate inositol triphosphate (IP3) and diacylglycerol (DAG). IP3 triggers the release of stored calcium and activates multiple downstream pathways [21]. DAG activates protein kinase C (PKC) along with its subsequent signaling cascades. In general, the PLC-γ pathway affects a variety of phenotypes including cell differentiation, transportation, and adhesion [40, 41].

#### STAT pathway

The STAT signaling pathway is also activated downstream of FGF. Activation of STAT1 in primary growth plate chondrocytes in response to FGF1 is necessary to inhibit proliferation [29]. STAT3 interacts with phosphorylated Tyr677 and the activated STAT pathways play a crucial role in regulating cellular gene expression [42].

## FGFs and OA

OA is a prevalent joint disease that primarily affects the knees, hips, hands, and spinal joints. Major clinical symptoms of OA include chronic joint pain, deformities, swelling, stiffness, and impaired joint function [43]. Normal articular cartilage consists of chondrocytes embedded in an ECM composed of collagen and proteoglycans [44]. In individuals with OA, disturbances often occur in chondrocyte proliferation and survival while collagen and proteoglycan levels in the cartilage matrix are reduced. Additionally, OA is characterized by enduring lesions in the articular cartilage along with subchondral bone sclerosis and osteophytosis [45]. The incidence of OA is affected by many factors such as environmental conditions, age, lack of exercise, obesity, genetic predisposition as well as trauma [46, 47]. Further investigation is required to elucidate the specific molecular mechanisms underlying OA. Once identified though these mechanisms can optimize existing diagnostic and treatment programs while proposing preventive measures to reduce excessive treatment.

The pathogenesis of OA is typically characterized by alterations in chondrocytes and the cartilage matrix, disrupting the homeostasis of articular cartilage and resulting in its destruction. The progression of radiological imaging and pain in OA is influenced by the development of synovitis and changes in synovial macrophage subtypes [48]. The FGF family plays a crucial role in maintaining the homeostasis of articular cartilage and IVDs, regulating various cellular processes such as growth, migration, differentiation, and survival. Disruptions in the FGF signaling pathway lead to modifications to chondrocyte proliferation and differentiation activity, as well as alterations to collagen fibers, proteoglycans, and other components of the cartilage ECM [49]. These pathological stimuli trigger macrophages to release pro-inflammatory factors that eventually result in cartilage deformation, injury, ectopic osteophyte formation, and other symptoms [50, 51]. In addition to the indirect stimulation of synovitis and changes in synovial macrophages through inflammatory substances, increased expression of FGF can directly accelerate these changes [52, 53]. In this review, we have identified FGF1, FGF2, FGF8, FGF9, FGF18, and FGF23 as being associated with both cartilage homeostasis maintenance and OA [54–70] (Table 1).

**Table 1:** Changes in the level of expression of members of the fibroblast growth factor (FGF) family in osteoarthritic cartilage

**FGF**	**Changes in osteoarthritis cartilage**	**Effect**	**Reference**
FGF1	Increased	Inhibit the expression of proteoglycan and type II collagen (Col-2) and matrix metalloproteinase 13 (MMP13)	[54, 55]
FGF2	Increased	Enhance the activation of Ras-mitogen-activated protein kinase pathways, leading to the induction of MMP13 and a disintegrin and metalloproteinase thrombospondin motifs-5 (ADAMTS-5)	[56–61]
FGF8	Increased	Reduce the content of sulfated glycosaminoglycan; Stimulate the expression of proMMP3 and prostaglandin E2 (PGE2); Interaction with interleukin-1, tumor necrosis factor-α	[62–64]
FGF9	Decreased	Promote proliferation of bone marrow stromal cells through the protein kinase B (Akt) pathways, maintains bone homeosta, and induces sry-related HMG-box gene 9 (SOX9) and Col-2	[65]
FGF18	Decreased	Promotes the growth and maturation of cartilage and the regeneration and repair of mature cartilage	[66–68]
FGF23	Increased	Increased in X-linked hypophosphatemia; Activation of p38-MAPK and Wnt signaling pathways affects synthesis, which leads to bone abnormalities by inhibiting mineralization and inducing chondrocyte apoptosis	[69, 70]

### FGF1

Increased levels of FGF1 have been observed in the chondrocytes of patients with OA [9]. FGF1 is secreted by the mesenchymal stromal cells and exerts paracrine effects to stimulate chondrocyte proliferation [54]. Treatment with FGF1 resulted in reduced levels of proteoglycan and collagen type II while inducing expression of matrix metalloproteinase 13 (MMP13), which has a catabolic effect on cartilage. FGF1 also binds to cellular communication network factor 2 (CCN2) protein and inhibits its transcription initiation level [55]. CCN2 is involved in the regeneration of damaged articular cartilage and the inhibition of OA progression [71]. Additionally, glycogen synthase kinase-3β (GSK3β) is essential for mediating the inhibitory response of chondrocytes to FGF. Activation of GSK3β by FGF1 signaling can be overridden by inhibiting GSK3β, leading to enhanced chondrocyte proliferation and differentiation both in cell culture and in vivo models [72].

### FGF2

**Fig. 1:**
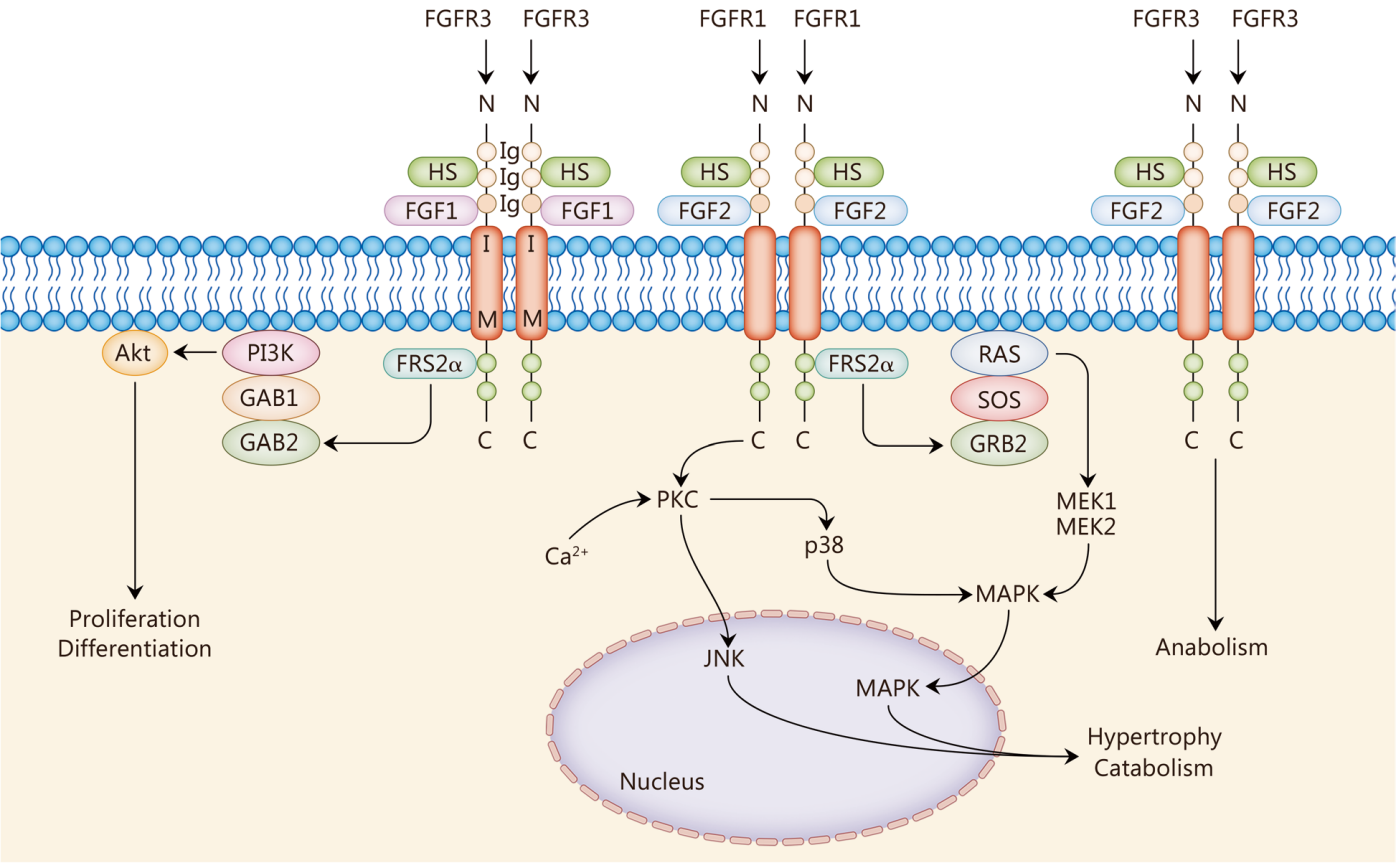
Fibroblast growth factors (FGFs) 1 and 2 in osteoarthritis (OA). FGF1 activates the PI3K-Akt pathway; FGF2 activates the Ras-MAPK pathway and PKC. The effects of each FGF-activating downstream signaling pathway on articular chondrocytes are shown. PI3K phosphoinositide 3-kinase, Akt protein kinase B, MAPK mitogen-activated protein kinase, PKC protein kinase C, GRB guanine nucleotide exchange factor, GAB GRB2-associated binding protein, HS heparan sulfates, FRS2α FGFR substrates 2α, SOS son of sevenless, MEK mitogen-activated extracellular signal-regulated kinase, FGFR3 fibroblast growth factor receptor 3, JNK c-Jun N-terminal kinase

FGF2 exerts both catabolic and anabolic effects on the homeostasis of human cartilage. When cartilage is overloaded or damaged, there is a significant increase in the release of FGF2, which activates a variety of signaling pathways including MAPK and downstream ERK, p38, and JNK. These 3 converge on an E twenty-six (ETS)-like transcription factor 1 (Elk-1), a transcription factor that can activate MMP13 [56, 57]. Similar to FGF1, FGF2 stimulates the upregulation of matrix-degrading enzymes MMP13 and a disintegrin and metalloproteinase thrombospondin motifs-5 (ADAMTS-5), leading to degradation of type II collagen (Col-2) in the cartilage matrix and downregulates of proteoglycan expression [58, 59]. Furthermore, FGF2 activates the downstream FGFR1-Ras/PKCδ-RAF-mitogen-activated extracellular signal-regulated kinase (MEK) 1/2-ERK1/2 signaling cascade [60]. FGF2 is also an activator of activator protein-1 (AP-1) and Runt-related transcription factor 2 (RUNX2), with the latter inducing ADAMTS-5 production. However, under normal conditions in cartilage tissue, FGF2 typically inhibits ADAMTS-5 activity thereby protecting articular cartilage. The ratio of FGFR1 to FGFR3 in the tissue likely determines the effect of FGF2 on cartilage [61]. In a previous study where either the expression ratio of FGFR1 to FGFR3 was decreased or the downstream pathway inhibition occurred, it resulted in reduced degradation of the cartilage matrix mediated by FGF2. This suggests that specific inhibitors targeting downstream signaling pathways activated by FGF2 could be used for prevention or treatment strategies against OA [56, 73]. However, some studies have confirmed that genes dependent on FGF2 (e.g., activin A and tissue inhibitor of metalloproteinase 1 (TIMP-1) exhibit neutral or protective effects in vivo [74, 75]; therefore, the duration and dosage considerations regarding blockade of FGF2 should be taken into account for future studies (Fig. 1).

### FGF8

FGF8 has the ability to bind to FGFRL1, FGFR2IIIc, FGFR3IIIc, and FGFR4 receptors, playing a key role in embryogenesis and morphogenesis [76]. It exerts a negative regulatory effect on osteogenic development. Co-expression of RUNX2 with SRY-related HMG-box gene 9 (SOX9) and Col-2 in ectopic cartilage highlights the differentiation of osteoprogenitor cells into chondrogenic cells during osteogenesis. FGF8 downregulates genes involved in ossification and bone mineralization while upregulating genes associated with proliferation, cartilage development, and cell fate commitment [10]. Abnormally elevated levels of FGF8 lead to the abnormal proliferation and differentiation of chondrocytes that affect osteogenesis in the cartilage. In an animal study conducted by Uchii et al. [77], it was found that FGF8 expression was significantly low in synovial cells and fibroblasts in normal joints but highly expressed in mechanically damaged joints, indicating that mechanical injury induces the expression of FGF8 in synovium and fibroblasts. FGF8 can also decrease the sulfated glycosaminoglycan content within the matrix, leading to increased production of proteases such as proMMP3 and prostaglandin E2 (PGE2) which subsequently degrade the cartilage matrix. Both FGF8 and FGFR3 are upregulated in hypertrophic chondrocytes [62]. Furthermore, FGF8 is associated with the inflammatory factors interleukin (IL)-1β and tumor necrosis factor-α (TNF-α). Under physiological conditions, IL-1 stimulates the release of neutral proteases and PGE2 from synoviocytes and articular chondrocytes [63], while TNF-α induces the production of various MMPs, such as MMP13, ADAMTS-5, and ADAMTS-7, as well as other inflammatory biomarkers [64]. These factors collectively contribute to articular cartilage changes and synovitis, playing a role in the degeneration of the articular cartilage matrix [78, 79]. Clinical studies have also supported the treatment of OA through inhibition of IL-1β and TNF [80, 81]. These two inflammatory factors are key downstream effectors in FGF8-induced degradation of the cartilage matrix [77].

### FGF9

Osteoblasts serve as the primary source of FGF9, which signals through FGFR3 in bone [82] to activate the Akt pathway, thereby stimulating the proliferation of bone marrow stromal cells, and maintaining bone homeostasis [83]. In chondrocytes affected by OA, FGF9 reduces oxidative stress, apoptosis, and mitochondrial dysfunction by promoting the nuclear translocation of nuclear factor erythroid 2-related factor 2 (Nrf2), activating the Nrf2/heme oxygenase-1 (HO1) signaling pathway. This attenuation leads to a decrease in interphalangeal narrowing and cartilage degradation in an OA mouse model [84]. Exogenous administration of FGF9 can inhibit the expression of matrix-degrading enzymes such as MMP13 in OA cartilage while promoting the expression of Col-2. However, it is important to note that exogenous FGF9 not only enhances the expression of SOX9 and Col-2 but also stimulates cell proliferation and contributes to or exacerbates osteophyte formation. These adverse effects can lead to a decline or loss of joint function and joint pain [65]. This phenomenon is attributed to FGF9’s stimulation of early chondrogenic differentiation, promotion of ECM production, and delayed terminal hypertrophy [85]. Furthermore, a recent study has shown that FGF9 can be used as a molecular marker for OA diagnosis [86]. Therefore, targeting FGF9 could be crucial for future OA diagnosis and treatment.

### FGF18

FGF18 is a growth factor that exerts its effects through heparin-binding polypeptides. It plays a role in the growth and maturation of cartilage in the musculoskeletal system as well as enhancing the regeneration and repair of mature cartilage [87, 88]. In an animal model of injury, FGF18 stimulated the formation and repair of cartilage [89]. In an in vitro model, FGF18 increased the synthesis of chondrocyte proteoglycan and Col-2, promoted chondrocyte proliferation, restored chondrocyte count, prevented chondrocyte apoptosis, and increased cartilage thickness [66–68]. FGF18 is believed to promote the chondrogenic activity of bone morphogenetic protein (BMP) by inhibiting the expression of noggin, a natural inhibitor of BMP [90]. A potential mechanism underlying the opposing effects of FGF2 and FGF18 on cartilage homeostasis may be attributed to their stimulation or suppression of noggin. The FGF18-FGFR3 axis promotes cartilage regeneration induced by BMP7 through antagonism with FGF2 [91]. Furthermore, it has been suggested that the proliferation of immature directional chondrocytes can be enhanced by FGF18 signaling through FGFR3. Additionally, it is speculated that FGF18 could serve as a growth factor preventing cartilage degradation and OA following surgery or mechanical cartilage injury. Both in vitro experiments and clinical trials have demonstrated that the recombinant human FGF18 (rhFGF18, sprifermin) treatment for knee OA patients stimulates the proliferation of chondrocytes and increases the ratio of Col-2 to type I collagen (Col-1). This leads to an enhanced production of chondrocyte ECM without any specific adverse effects [68, 92, 93]. Based on multiple experimental studies, rhFGF18 is anticipated to be a promising disease-modifying drug for OA treatment [93–97].

### FGF23

**Fig. 2:**
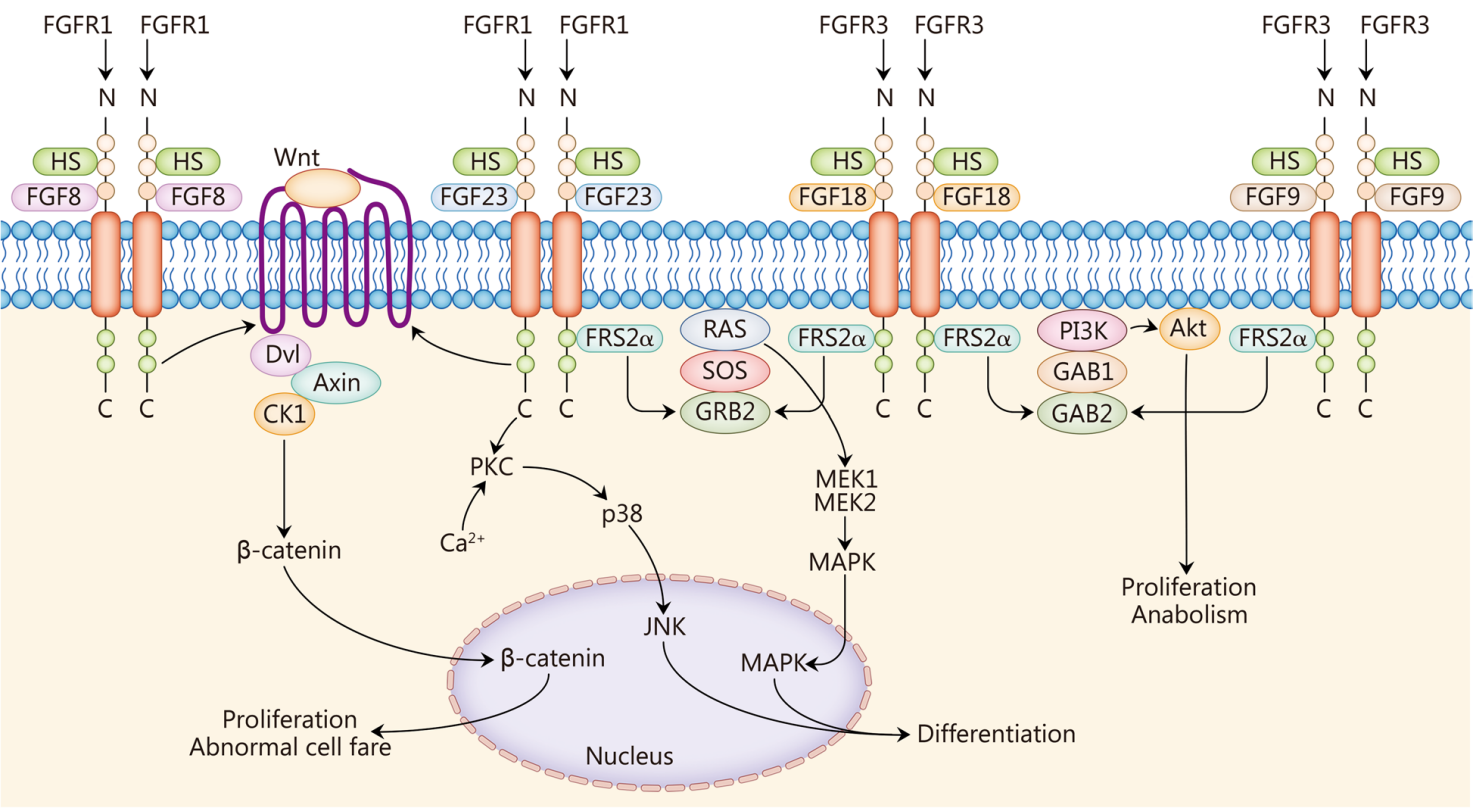
Fibroblast growth factors (FGFs) 8, 9, 18, and 23 in osteoarthritis (OA). FGF8 and FGF23 activate the Wnt/β-catenin pathway; FGF9 and FGF18 activate the PI3K-Akt pathway; FGF18 and FGF23 activate the Ras-MAPK pathway. The effects of each FGF-activating downstream signaling pathway on articular chondrocytes are shown. Dvl dishevelled, Axin axis inhibition, CK1 casein kinases 1, HS heparan sulfates, FRS2α FGFR substrates 2α, SOS son of sevenless, GRB guanine nucleotide exchange factor, GAB GRB2-associated binding protein, PKC protein kinase C, JNK c-Jun N-terminal kinase, MEK mitogen-activated extracellular signal-regulated kinase, MAPK mitogen-activated protein kinase, PI3K phosphoinositide 3-kinase, Akt protein kinase B

FGF23 levels are significantly elevated in patients diagnosed with X-linked hypophosphatemia (XLH) [69]. XLH can affect various physiological aspects, particularly bone growth, including OA, osteomalacia, bone abnormalities, bone pain, and other symptoms [69]. The presence of phosphate in bone tissue can lead to an increase in serum FGF23. Excessive FGF23 levels result in hypophosphatemia which causes bone abnormalities by inhibiting mineralization and inducing apoptosis of hypertrophic chondrocytes. Insufficient mineralization in newly formed bone leads to the accumulation of osteoids, reduced bone strength, and joints and long bone abnormalities. Furthermore, the upregulation of FGF23 as a negative regulator of chondrogenesis prompts premature termination of the proliferative state among chondrocytes leading them into a hypertrophic state. Elevated expression of FGF23 in OA chondrocytes activates the expression of RUNX2 that subsequently upregulates MMP13 expression [70]. In osteocytes, p38-MAPK and PKC signaling affect the synthesis of FGF23. These effects partly depend on the nuclear factor kappa-light-chain-enhancer activated B cells (NF-κB) activity [98, 99]. A mouse model exhibiting high expression of high-molecular-weight FGF2 demonstrated increased expression of FGF23 in articular cartilage along with activation of the classical Wnt signaling pathway [100]. This indicates that there is a close relationship between FGF23 and the signaling pathways associated with other subtypes within the FGF family, highlighting its ability to regulate Wnt/β-catenin signal transduction for facilitating chondrocyte differentiation. Consequently, it is imperative to consider FGF23 as a potential risk factor in the development of OA and cartilage disorders (Fig. 2).

## FGFs and cartilage ossification

In addition to regulating the proliferation of cartilage and degradation of ECM, FGFs also play a role in cartilage ossification. The signaling pathway of FGF/FGFR3 regulates bone growth during the process of cartilage osteogenesis. FGFR3 in periosteal cells controls chondrocyte hypertrophy and fibrosis, serving as a crucial regulatory factor in the transformation from cartilage to bone [101]. Different FGFs have varying effects on cartilage osteogenesis. Treatment with FGF2 led to the thickening of the cartilage growth plate, while hypertrophic chondrocytes exhibit flattened morphology with irregular arrangement in the lowermost part of FGF2-treated cartilage tissue. Additionally, FGF2 inhibits angiogenesis and endochondral ossification at the growth plate [102].

The combination of FGF9 and FGFR3 activates the downstream ERK1/2 signaling pathway. The FGF signaling mediated by the ERK1/2 pathway enhances SOX9 expression in chondrocytes [103], thereby increasing the chondrocytes density and promoting cartilage formation. Moreover, elevated levels of FGF9 promote longitudinal bone growth. Thus, combined signaling through FGF9 and FGFR3 results in increased thickness of cartilage, accelerated osteogenesis within the cartilaginous tissue, and enhanced length and width development in long bones [104].

FGF18 regulates cartilage osteogenesis through the activation of FGFR1-3. In a previous study, deletion of FGF18 led to delayed ossification, shorter bone length, and abnormal bone shape in mice, resulting in a lethal phenotype in FGF18 knockout mice [105]. Furthermore, FGF18 inhibits chondrocyte proliferation and differentiation in the growth plate of rodents but induces proliferation and differentiation in articular cartilage [106]. This generally leads to the thickening of cartilage growth plates and an increase in hypertrophic chondrocytes. Conversely, during chondrocyte development and in adult mammalian cartilage, FGF18 promotes chondrocyte proliferation and differentiation via FGFR3. It has been reported that FGF18 promotes the differentiation of hypertrophic chondrocytes into osteoblasts by activating FGFR1 in hypertrophic chondrocytes and FGFR2 in the perichondrium and trabecular bone [107]. However, most studies have indicated that FGF18 promotes cartilage formation while inhibiting osteoblast differentiation [108–110]. Previous studies confirmed that FGF18 activates JNK1, which phosphorylates BCL2 [111]. Additionally, FGF18 regulates the activity of the phosphatidylinositol 3-kinase catalytic subunit type 3 (VPS34)-beclin-1 complex to induce autophagy, while regulating ECM remodeling through chondrocyte autophagy in the cartilage growth plate [111].

## FGFRs and OA

The expression of FGFR1-3 in articular cartilage is observed, with significantly higher levels of FGFR1 and FGFR3 compared to FGFR2. Inhibition of FGFR3 leads to a significant increase in the expression of FGFR1, which subsequently stimulates the catabolism of articular cartilage [56, 112]. During the pathogenesis of OA, an imbalance in the expression ratio between FGFR1 to FGFR3 may promote chondrocyte decomposition, and apoptosis, and hinder anabolism.

### FGFR1

FGFR1 exhibits high expression in human knee chondrocytes. FGF2 binds to FGFR1, triggering the downstream Ras-Raf-MEK1/2-ERK1/2 signaling pathway and subsequently activating RUNX2, ETS-like transcription factor 1 (ELK1), and other transcription factors. This activation leads to upregulation of downstream genes, promotion of chondrocyte catabolism, and inhibition of proteoglycan formation. The FGF2-mediated FGFR1-ERK pathway negatively regulates FGFR3. Activation of FGFR1 by FGF2 induces chondrocyte hypertrophy. Imbalance in chondrocyte proliferation and survival, as well as abnormal hypertrophy of chondrocytes, are critical processes influencing articular cartilage during OA progression [113]. Furthermore, FGFR1 plays a significant role in cartilage injury mediated by IL-1β and other inflammatory factors. Specifically, blocking FGFR1 effectively alleviated cartilage catabolism [114]. R1-P1, a peptide inhibitor targeting FGFR1, modulates the ERK1/2 pathway while reversing IL-1β-induced loss of proteoglycans and Col-2. It also attenuates the expression of MMP13 and significantly reduces the destruction of the articular cartilage [115]. These findings suggest that an inhibitor targeting FGFR could potentially serve as a therapeutic drug for OA treatment. Furthermore, in mice with FGFR1 knockout, reduced MMP13 expression was observed along with decreased cartilage degeneration, leading to delayed OA progression [116].

### FGFR3

The expression of FGFR3 is higher in healthy articular cartilage but significantly lower in patients with OA. Mutations in human FGFR3 lead to abnormal bone development, and this is also observed in FGFR3-knockout mice [117, 118]. Several studies have shown that FGFR3 plays a crucial role in maintaining articular cartilage homeostasis. It also promotes the transformation of cartilage to bone [101, 119], and the reduced expression of FGFR3 may be responsible for the abnormal bone development seen in OA. Deletion or reduced expression of FGFR3 results in an increase in MMP13 and type X collagen in the cartilage, thereby enhancing DMM-induced cartilage degeneration [4, 119].

Through FGFR3, the downregulation of chondrocyte proliferation by STAT1 ensures coordinated bone development and morphogenesis via FGFR. In mutant or abnormally expressed FGFR3, there is an overexpression of STAT1 and STAT5 in pre-hypertrophic chondrocytes, indicating a dual role of FGFR3-STAT signaling in the regulation of cartilage development [120, 121]. The activation of the RUNX2 pathway occurs through the PI3K-Akt pathway and induces chondrocyte maturation and differentiation via FGFR3 [122].

In human joints, FGF9 and FGF18 exhibit high specific affinities for FGFR3 [85], leading to downstream activation of RAS-MAPK and PI3K-Akt pathways, stimulation of Fox transcription factor, promotion of articular cartilage synthesis, promotion of articular cartilage synthesis and matrix production, and enhancement of cell activity and repair [87, 89]. Additionally, FGF2 binds to FGFR3 to promote cartilage anabolism, suggesting its dual roles in cartilage development [123]. FGFR3 serves as a crucial factor for articular cartilage protection, with its expression levels reflecting the extent of cartilage damage. Therefore, targeting the FGFR3 pathway may present a novel approach for OA. Currently, available therapeutic strategies for OA mainly involve nonsteroidal anti-inflammatory drugs and opioid analgesics, as no disease-modifying OA drugs have been approved yet. Among the FGF family members, FGF9 and FGF18 hold potential as targets for emerging molecular drugs. Furthermore, antibodies against the pathways of FGF1, FGF2, and FGF8 exhibit promising prospects in alleviating pain and mitigating cartilage damage. In the research and development of emerging molecular drugs, our focus should not only be on reducing pain and preventing further cartilage damage but also on regenerating damaged joint cartilage and restoring normal joint function. Compared to traditional medications, emerging molecular drugs demonstrate more pronounced effects, such as reduced cartilage damage and fewer side effects; thus, they offer broad prospects for research and application.

At present, the primary focus of the research and development in molecular-targeted drugs lies on FGF9, FGF18, and FGFR3. Other components within the FGF signaling pathway play a lesser role in the development of bones and joints as well as the pathogenesis of related diseases, necessitating further exploration. Specifically, there is a need for additional investigation into the interactions between members of the FGF family, particularly those associated with OA, during cartilage, bone, and joint development processes. Further research is needed to understand the involvement of FGFs in OA progression and develop targeted molecular therapies for OA and related cartilage disorders. This will enhance existing diagnosis and treatment schemes, while reducing pain levels, facilitating damaged cartilage repair, and restoring joint function.

## Research and development of new drugs related to FGFs in OA

### FGF18 and sprifermin

As previously mentioned, FGF18 stimulates cartilage proliferation and promotes cartilage maturation. Among the development of FGF-related OA drugs, those based on FGF18 have been extensively studied. Notably, sprifermin has completed phase II clinical trials and demonstrated efficacy in several non-clinical models as well as in three clinical studies [108, 124, 125]. Sprifermin is a truncated form of FGF18 consisting of 170 amino acids with the signal sequence and 11 C-terminal acids removed. It stands out as one of the few drugs that effectively enhances cartilage proliferation and increases cartilage thickness. FGF18 is a growth factor for mature human chondrocytes and their progenitor cells, as well as in chondrocytes derived from various animal species including rats, rabbits, sheep, pigs, cows, dogs, and horses [66, 67, 126]. This indicates that FGF18 plays a highly conserved role in maintaining cartilage homeostasis among mammals. In vitro studies have shown that sprifermin significantly increases cell proliferation in a dose-dependent manner at concentrations of 0.1 – 1000 ng/ml with a median effect concentration (EC50) value of approximately 10 ng/ml [66]. It also increased the expression of Col-2 at concentrations of 0.1 – 100 ng/ml, while reducing Col-1 expression, and promoting SOX9 expression in a dose-dependent manner. However, when used at concentrations exceeding 1000 ng/ml, the proliferative effect on cartilage gradually weakens [66]. Compared to insulin-like growth factor 1/2, BMP7, and other growth factors, Sprifermin exhibits superior effects on promoting cell proliferation [67, 126]. Under cyclic sprifermin treatment, there was a significant increase in the expression of COL2A1 and ACAN, and the proportion of Col-2/Col-1 was significantly higher than that achieved with other growth factors [67]. Sprifermin not only promotes cell proliferation but also enhances cartilage regeneration by producing a more normal hyaline cartilage matrix [66]. Furthermore, sprifermin effectively stimulates cartilage proliferation in various animal models. In a rat model, weekly injections of 3 – 10 µg rhFGF18 effectively alleviated cartilage degeneration and increased cartilage matrix activity [127]. Additionally, intra-articular injection of rhFGF18/sprifermin has been shown to reduce injury-related cartilage loss in dogs, rabbits, sheep, and other animal models [106, 128, 129]. In terms of safety and systemic exposure levels for promoting cell proliferation and matrix synthesis, intra-articular delivery of sprifermin has demonstrated favorable results [130]. However, excessive frequency or high doses of intra-articular sprifermin injections may stimulate joint tissues prone to inflammation and injury. A phase I clinical trial [131] involving 73 patients scheduled for total knee replacement (TKR) due to OA divided them into 3 groups (single dose/multiple doses/placebo). Each dose of sprifermin resulted in serious adverse reactions without significant differences among the groups. Although administration of 300 µg sprifermin commonly causes an inflammatory reaction, it does not necessitate immediate termination. The histological and metabolic findings from this phase I study indicated that sprifermin promoted cell proliferation and matrix synthesis, while imaging revealed improvement in cartilage quality. Notably, the high-dose groups receiving either 100 µg or 300 µg had lower probabilities for TKR events compared to the lower-dose groups, despite meeting the inclusion criteria for scheduled TKR surgery. In a phase II clinical trial, patients were treated with different concentrations (30 and 100 ng/ml) and frequencies (q6mo/q12mo) of rhFGF18. The total treatment duration was up to 1.5 years, followed by a 3.5-year follow-up period, resulting in a 5-year study. Similar to the findings obtained from in vitro experiments, the promotion of cartilage proliferation was dose-dependent. It was observed that a high-concentration dosage (100 ng/ml) administered at a high-frequency interval (q6mo) effectively increases the thickness of cartilage. However, there was no significant difference in cartilage thickness between the groups receiving sprifermin at doses of 30 µg every 6 or 12 months and placebo. Sprifermin primarily induces cartilage thickening as its main effect, while arthralgia is identified as its primary adverse reaction based on trial results. Compared with other growth factors, sprifermin demonstrates superior efficacy in promoting cartilage growth and proliferation [67]. The clinical trial for OA involving sprifermin revealed its effectiveness in promoting cartilage proliferation, increasing cartilage thickness, improving metabolic activity, and relieving pain. Nevertheless, further investigation is required to assess its clinical effects and safety profile. Sprifermin successfully achieved its primary endpoint of inducing changes in cartilage thickness during the FORWARD phase II trial [68]. A post-hoc analysis conducted on this phase II trial demonstrated symptomatic benefits associated with sprifermin treatment [93].

### FGF2

Research on the treatment of OA by targeting FGF2 has primarily focused on the utilization of recombinant adeno-associated virus (rAAV) to induce overexpression of FGF2. In vitro studies have demonstrated that rAAV-mediated overexpression of FGF2 promotes prolonged chondrocyte proliferation. Furthermore, in vivo animal experiments have shown that increased expression of FGF2 enhances repair, filling, architecture, and cell morphology in osteochondral defects [132]. Additionally, sustained high levels of FGF2 expression have been found to facilitate the restoration of damaged cartilage tissue [133]. Current research suggests that therapeutic delivery of human FGF2 through direct administration of rAAV can effectively enhance long-term osteochondral repair. Moreover, gene therapy mediated by rAAV holds promising potential for future clinical applications in cartilage regeneration.

Currently, multiple nano-delivery systems are utilized for the targeted delivery of nano-drug delivery systems to the FGF family and FGF2 [134]. Electrospun chitosan fiber networks decorated with heparin-containing polyelectrolyte complex nano-particles can effectively adsorb FGF2 and form a sustained-release system for growth factors. Nano-carriers enable the gradual release of FGF2 throughout 30 d, thereby playing a long-term role in promoting the surrounding tissues’ proliferation. Low-molecular-weight heparin/protamine nano-particles can encapsulate FGF2, ensuring its stability in the nano-delivery system, and extending the biological half-life of FGF2 [135]. Incorporating FGF2 into a nano-material scaffold enhances the biocompatibility of the material while significantly promoting osteogenic differentiation of bone marrow stromal cells (BMSC). The utilization of FGF-modified nanomaterial scaffolds holds great potential in orthopedic diseases involving bone defects or loss [136, 137]. At present, research and development efforts focused on FGF-related nano-drug delivery systems for orthopedic diseases aim to exploit the proliferative effects of FGF2 on BMSCs as well as its ability to induce osteogenic differentiation. This approach involves developing biological scaffolds or drug delivery systems loaded with FGF2 to facilitate bone defect repair and address osteoporosis.

### Combined use of growth factors

Sequential exposure to FGF2, 9, and 18 enhances chondrogenesis and differentiation. During the early stages of chondrogenesis and expansion, treatment with FGF2 upregulates the expression of FGFR1 and SOX9, thereby promoting early chondrogenesis. Subsequently, during chondrogenic induction, there is a gradual decrease in FGFR1 expression leading to a weakened effect on both chondrogenesis and differentiation.

### FGF9 and FGF18

Both FGF9 and FGF18 can combine with FGFR3 to enhance cartilage anabolism. However, in the absence of early-stage amplification or expression of FGF2, the expression levels of FGF9 and FGF18 remain low. During late-stage amplification and expression of FGF2, elevated levels of FGF9 and FGF18 significantly enhance cartilage anabolism through FGFR3. Nevertheless, their anabolism-enhancing effects are not evident when expressed during the early stages of FGF2 amplification [85]. This study demonstrates the diverse roles played by different FGFs in chondrocyte proliferation and differentiation. The combined utilization of multiple FGFs can more effectively promote cartilage proliferation, differentiation, and anabolism while reducing adverse reactions associated with individual use.

In addition to the combination of FGFs, the concurrent utilization of FGFs in conjunction with other growth factors, such as TGF-β, insulin-like growth factor 1 (IGF-1), and PDGF, can significantly improve cartilage proliferation while exerting minimal effect on chondrocyte genetic stability and tumorigenicity [138]. However, further investigation is warranted to assess the clinical efficacy and safety of this approach.

## FGFs and IVDD

**Fig. 3:**
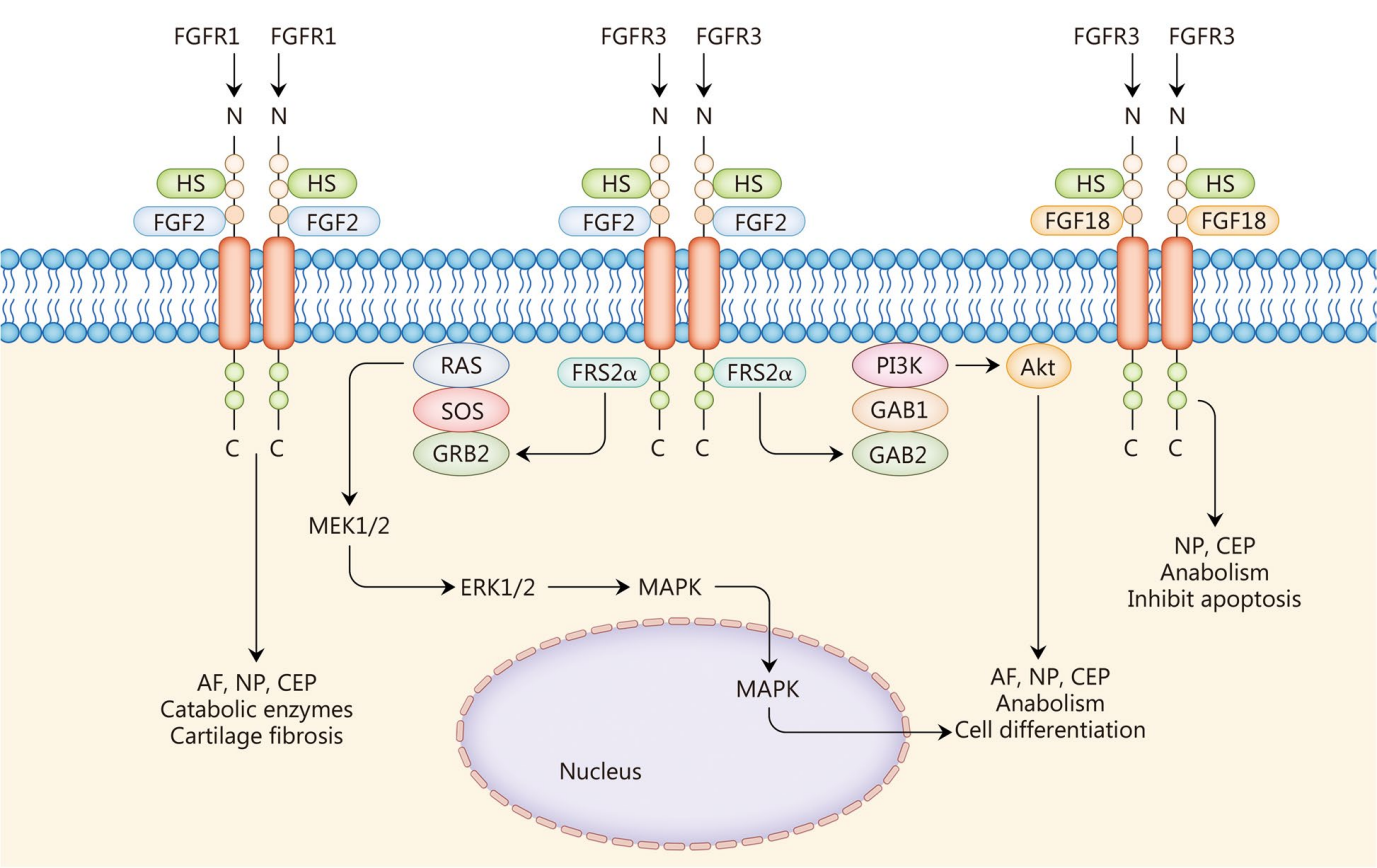
Fibroblast growth factors (FGFs) in intervertebral disc degeneration (IVDD). FGF2 promotes the catabolism of AF, NP, and CEP through FGFR1, and fibrosis of IVD cartilage. FGF2 works through FGFR3 to activate downstream Ras-MAPK and PI3K-Akt signaling pathways to promote anabolism and cell differentiation in various tissues of IVDs. FGF18 promotes tissue synthesis and apoptosis via FGFR3. AF annulus fibrosus, NP nucleus pulposus, CEP cartilaginous endplate, ERK extracellular signal-regulated kinase, MAPK mitogen-activated protein kinase, HS heparan sulfates, FRS2α FGFR substrates 2α, SOS son of sevenless, GRB guanine nucleotide exchange factor, GAB GRB2-associated binding protein, PI3K phosphoinositide 3-kinase, Akt protein kinase B

IVDD is influenced by various factors, including genetics, aging, and mechanical damage. IVDs consist of the NP, cartilaginous end-plates (CEP), and annulus fibrosus (AF), which predominantly comprises angular type 1 collagen with internally dispersed fibroblasts and chondrocytes that overlap. Compared with normal IVDs, degenerative IVDs exhibit numerous histological changes such as NP fibrosis, fissure formation, and cell clusters. The AF fiber structure is destroyed and vascularized with cracks formed. CEPs display thinning, mineralization, microfractures, and bone sclerosis among other abnormalities [139]. At the molecular level, degenerative IVDs show decreased proteoglycans and Col-2 but increased Col-1 [140]. Additionally, IVDD is accompanied by an increase in inflammatory factors and activation of inflammatory pathways where IL-1β and TNF-α can activate catabolic molecules, thereby leading to increased MMPs, ADAMTS, COX2, and other decomposing proteins [141]. Abnormal changes occur in FGF signaling during the development of IVDD. The roles of each FGF in its pathogenesis are discussed below (Fig. 3, Table 2).

**Table 2:** Changes in the level of expression of members of the fibroblast growth factor (FGF) family in disease

**FGF**	**Changes**	**Effect**	**Reference**
Intervertebral disc disease			
FGF2	Increased	Low concentration of FGF2 activates the mitogen-activated extracellular signal-regulated kinase (MEK)-extracellular signal-regulated kinase (ERK) and phosphatidylinositol-3-kinase - protein kinase B (Akt) pathways and promotes the proliferation of NP and AF cells; Promote fibrocartilage synthesis	[142, 143]
FGF18	Decreased	Promote cartilage synthesis; Inhibit cartilage catabolism	[11, 144]
Osteoporosis			
FGF2	Decreased	Promote parathyroid hormone -mediated bone anabolic metabolism through the Wnt/β-catenin signaling pathway and maintains calcium phosphate homeostasis to promote bone growth, development, and fracture healing	[145]
FGF19	Decreased	Regulate glucose and lipid metabolism and bile acid metabolism, and activate the Wnt/β-catenin signaling pathway, promote osteoblast differentiation, inhibit osteoclast generation	[146, 147]
FGF21	Increased	Improve glucose and lipid metabolism, indirectly affecting bone mass	[8, 148]
FGF23	Increased	Regulate calcium and phosphorus metabolism and reduce bone formation	[149, 150]
Sarcopenia			
FGF2	Increased	Increase muscle mass and promote intramuscular adipose tissue	[151–153]
FGF19	Decreased	Improve muscle glucose and lipid metabolism and promote muscle growth	[154, 155]
FGF21	Increased	Promote muscle cell growth by activating the ERK1/2 signaling pathway and ribosomal protein S6 kinase 1 (S6K1) and improve systemic metabolism and reduce muscle mass	[156, 157]
FGF23	Increased	Promote aging of muscle and stem cells	[158, 159]

### FGF2

Similar to the effect of FGF2 on chondrocytes in OA, the role of FGF2 in IVDs and the pathogenesis of IVDD remains a subject of controversy. Some studies have demonstrated that FGF2 promotes mitosis and substance synthesis during IVD [142, 143]. As an autocrine growth factor, low concentrations of FGF2 activate the MEK-ERK and PI3K-Akt pathways, thereby inducing DNA synthesis and promoting the proliferation of NP and AF cells [160]. Additionally, FGF2 can maintain tissue sensitivity to TGF-β, enhance sulfated proteoglycan (PG) synthesis, reduce ACAN turnover, and facilitate NP cell differentiation [161, 162]. However, several studies have indicated that FGF2 promotes IVD catabolism. Its catabolic effects are primarily mediated by FGFR1-induced upregulation of catabolic enzymes, leading to increased fibrocartilage content in cartilage while decreasing cartilage mass. Furthermore, it elevates levels of catabolic enzymes in the ECM while inhibiting the anabolic effects of IGF-1 and BMP7 [161, 163]. Melrose et al. [164] found varying expression levels of FGF2 at different stages which often account for its diverse functions. Li et al. [91] proposed a dose-dependent relationship between total PG accumulation, PG synthesis, and elevated expression levels of FGF2 resulting in decreased PG synthesis. In summary, the activity of FGF2 in IVDD is similar to that in OA. Although it plays a crucial role in the growth and differentiation of cartilage, an excessive amount of FGF2 can lead to pathological changes in cartilage tissue. However, there is still ongoing debate regarding the specific contribution of FGF2 to joint degeneration.

### FGF18

Studies on the expression of FGF18 in IVDD are still in their early stages. Similar to articular cartilage, FGF18 plays a protective role in IVD cartilage by promoting anabolism and inhibiting the expression of proteases such as MMP3 and ADAMTS-5. Additionally, FGF18 can inhibit the apoptosis of NP cells, thereby providing further protection for the IVD. FGF18 increases the expression of Col-2 and CA12 in NP cells while decreasing degeneration in vertebral joint NP [11, 144]. Currently, research related to FGF18 primarily focuses on cartilage and NPs rather than other tissues like AF. However, it should be noted that FGF18 has not yet been utilized as a drug for IVDD.

## FGFs and OP

**Fig. 4:**
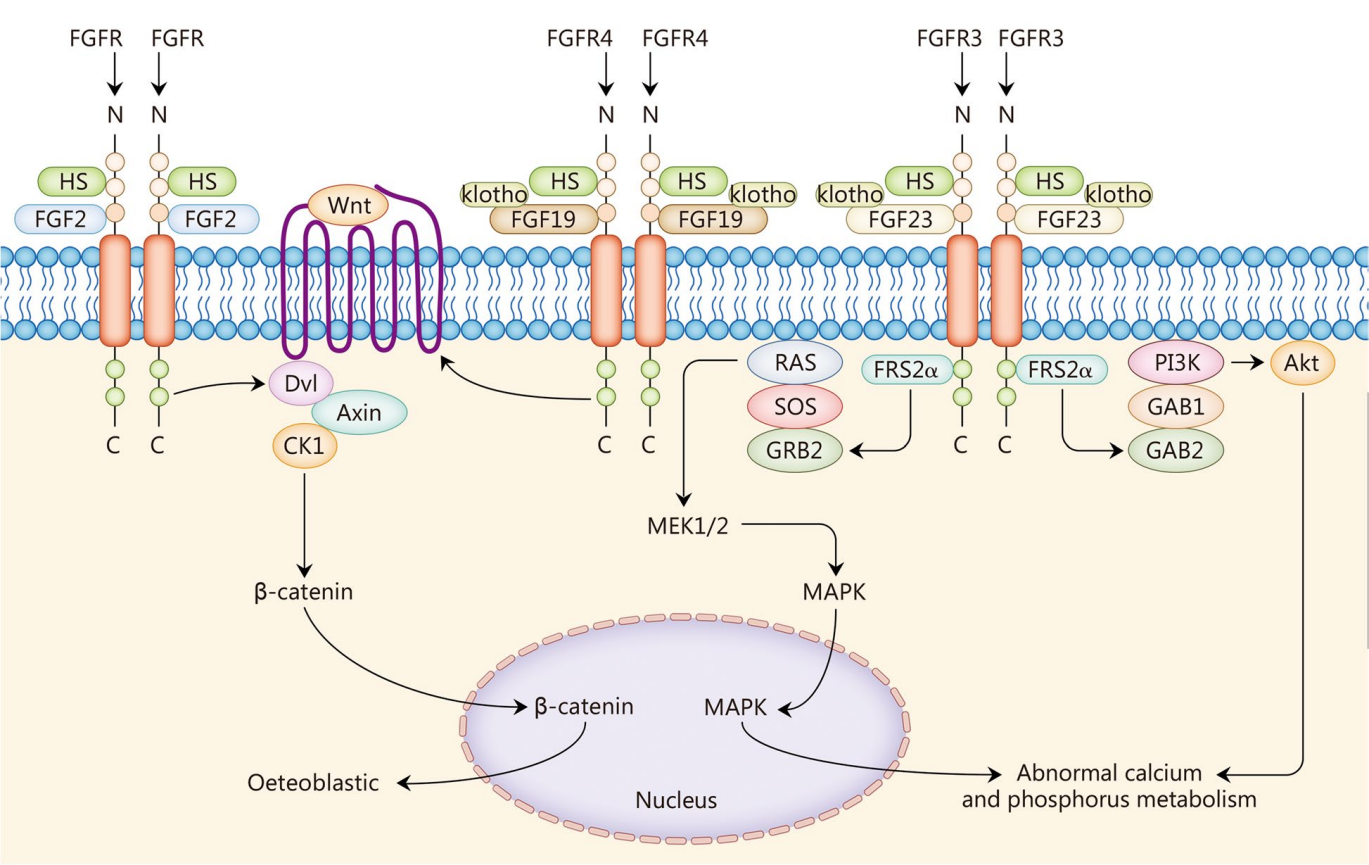
Fibroblast growth factors (FGFs) in osteoporosis (OP). FGF2 and FGF19 promote osteogenesis through the Wnt-β-catenin signaling pathway. FGF23 binds to FGFR3 to affect osteoblast metabolism through the RAS-MAPK and PI3K-Akt signaling pathways, as well as calcium and phosphate metabolism. It also affects calcium and phosphate metabolism by influencing hormone secretion and cell metabolism. Dvl dishevelled, Axin axis inhibition, CK1 casein kinases 1, MEK mitogen-activated extracellular signal-regulated kinase, MAPK mitogen-activated protein kinase, PI3K phosphoinositide 3-kinase, Akt protein kinase B, GRB guanine nucleotide exchange factor, GAB GRB2-associated binding protein, HS heparan sulfates, FRS2α FGFR substrates 2α, SOS son of sevenless

The pathogenesis of OP involves disruption of bone metabolism, leading to a systemic skeletal disease. Fractures and other bone disorders associated with OP are increasingly prevalent in women aged over 55 years and men aged over 65 years. OP is characterized by decreased bone mass and density, as well as micro-architectural deterioration of bone tissue. The pathological process of OP can be classified into reduced bone synthesis, increased bone absorption, and destructive changes [165]. The FGF family of proteins plays essential roles in the regulation of bone formation, repair, regeneration, angiogenesis, and metabolism [145]. FGF2 and other factors also influence and regulate musculoskeletal signal crosstalk, maintain musculoskeletal homeostasis, and delay degeneration [8, 145–150] (Fig. 4, Table 2).

### FGF2

FGF2 plays a crucial role in regulating bone regeneration and cartilage differentiation; it is expressed in osteoblasts, stored in the ECM, and serves as a key regulator of osteoblast function. Disruption of FGF2 expression can lead to decreased osteoblast replication and impaired new bone formation [166]. FGF2 can also facilitate bone anabolism mediated by parathyroid hormone (PTH) through the Wnt/β-catenin signaling pathway [167]. Additionally, FGF2 promotes bone growth, development, and fracture healing via the Wnt signaling pathway, BMP2 activation, and maintenance of calcium phosphate homeostasis [168]. With promising therapeutic potential, FGF2 holds prospects for promoting osteogenesis, fracture healing, and regulation of bone calcium-phosphorus balance. Furthermore, it can be utilized as a coadjuvant in the existing treatment methods. For example, administering FGF2 during intermittent PTH treatment may be beneficial [169].

### Endocrine FGFs

The FGF19 subfamily, also known as endocrine FGFs, consists of FGF19, FGF21, and FGF23. In comparison to other members of the FGF family, endocrine FGFs exhibit a relatively low affinity for FGFR. The binding of the cofactor klotho to FGFR is essential for the interaction between endocrine FGFs and this receptor. Endocrine FGFs play crucial roles in regulating calcium-phosphorus balance, bile acid secretion, as well as glucose and lipid metabolism. They are closely associated with conditions such as atherosclerosis, mitochondrial diseases, musculoskeletal diseases, and various other tissue-related ailments [170].

#### FGF19

FGF19 is secreted by the ileal epithelial cells and forms a complex with FGFR4 and β-klotho. It primarily lies in regulating hepatic glycolipid and bile acid metabolism [146]. A previous study observed lower levels of bile acid and FGF19 in postmenopausal women with OP compared to normal women [171]. Changes in FGF19 expression or abnormal bile acid metabolism may significantly contribute to the development of OP. Guo et al. [172] found that FGF19 can ameliorate obesity-induced bone loss caused by activating the Wnt/β-catenin signaling pathway, promoting osteoblast differentiation, and inhibiting osteoclastogenesis through osteopontin. Moreover, it has been suggested that FGF19 can modulate BMD in vivo by regulating bile acid balance and lipid metabolism [146, 171].

#### FGF21

FGF21 exhibits high expression in the liver, adipose tissue, and muscle. In the context of muscle tissue, increased FGF21 expression enhances the uptake and utilization of glucose and improves lipid metabolism [148, 173]. FGF21 is closely related to fat metabolism, muscle diseases, and mitochondrial diseases. However, further investigation is required to understand the metabolic relationship between FGF21 and bone tissue. Experimental studies on rats have demonstrated that exogenous administration of FGF21 leads to increased bone absorption, reduced bone formation, and decreased BMD [174, 175]. Some studies suggest a negative correlation between serum FGF21 and BMD [176, 177], whereas others indicate no significant association between FGF21 expression and BMD. The influence of BMD by FGF21 or by abnormalities in the metabolism of glucose and lipids due to liver or muscle tissue dysfunction remains unconfirmed [178, 179]. Indirectly impacting bone mass through regulation of glucose and lipid metabolism as well as secretion of factors related to muscle tissue function characterizes the role of FGF21. Nevertheless, further research is needed to determine whether it directly affects bone tissue along with its specific impact on BMD within pathological conditions affecting special populations.

#### FGF23

FGF23 is synthesized by osteoblasts under normal physiological conditions. It binds to FGFR through klotho and plays a regulatory role in the metabolism of vitamin D, phosphate, and other minerals in the body [180]. The metabolic regulation function of FGF23 involves multiple cellular signaling pathways, including the MAPK and PI3K signaling pathways, as well as its impact on PTH signaling. IGF-1 directly and indirectly regulates calcium and phosphorus metabolism in the body [17, 149, 181]. In addition to its dependence on klotho, FGF23 can also independently modulate local bone mineralization through FGFR3 [150]. Overall, FGF23 exerts various effects on bone mineral metabolism in vivo. Overexpression of FGF23 leads to excessive phosphate consumption, hypophosphatemia, reduced vitamin D levels, and decreased BMD.

The expression of FGF23 is upregulated in patients with OP [182]. FGF23-induced bone loss is a consequence of mineral and bone disorders associated with chronic kidney disease (CKD). FGF23 suppresses the production and secretion of PTH while inhibiting the synthesis of 1,25(OH)2D3 in the kidneys [183]. Intermittent use of PTH in the early stages of CKD may alleviate phosphorus retention, thereby potentially elevating FGF23 levels in CKD patients with coexisting osteoporosis [184]. In bone, FGF23 directly interacts with FGFR3 in a klotho-independent manner and inhibits osteoblast activity. It also forms a complex with FGFR3 through klotho, downregulating tissue-nonspecific alkaline phosphatase (TNAP), consequently reducing bone mineralization [185]. By influencing bone tissue directly and regulating calcium, phosphorus, and mineral metabolism through endocrine signaling pathways, FGF23 reduces the synthesis of bone minerals while enhancing their decomposition. Numerous studies have explored the specific role and regulation mechanisms underlying FGF23 in OP. In future investigations, targeting FGF23 could prove essential for preventing, monitoring, and treating OP [186, 187]. Nevertheless, it should be noted that apart from its impact on bones, FGF23 can also affect other organs such as the heart, kidney, and parathyroid glands. Therefore when investigating its mechanism or developing drugs related to FGF23 action, it is crucial to consider its effects on these organs to minimize potential adverse reactions.

## FGFs and sarcopenia

**Fig. 5:**
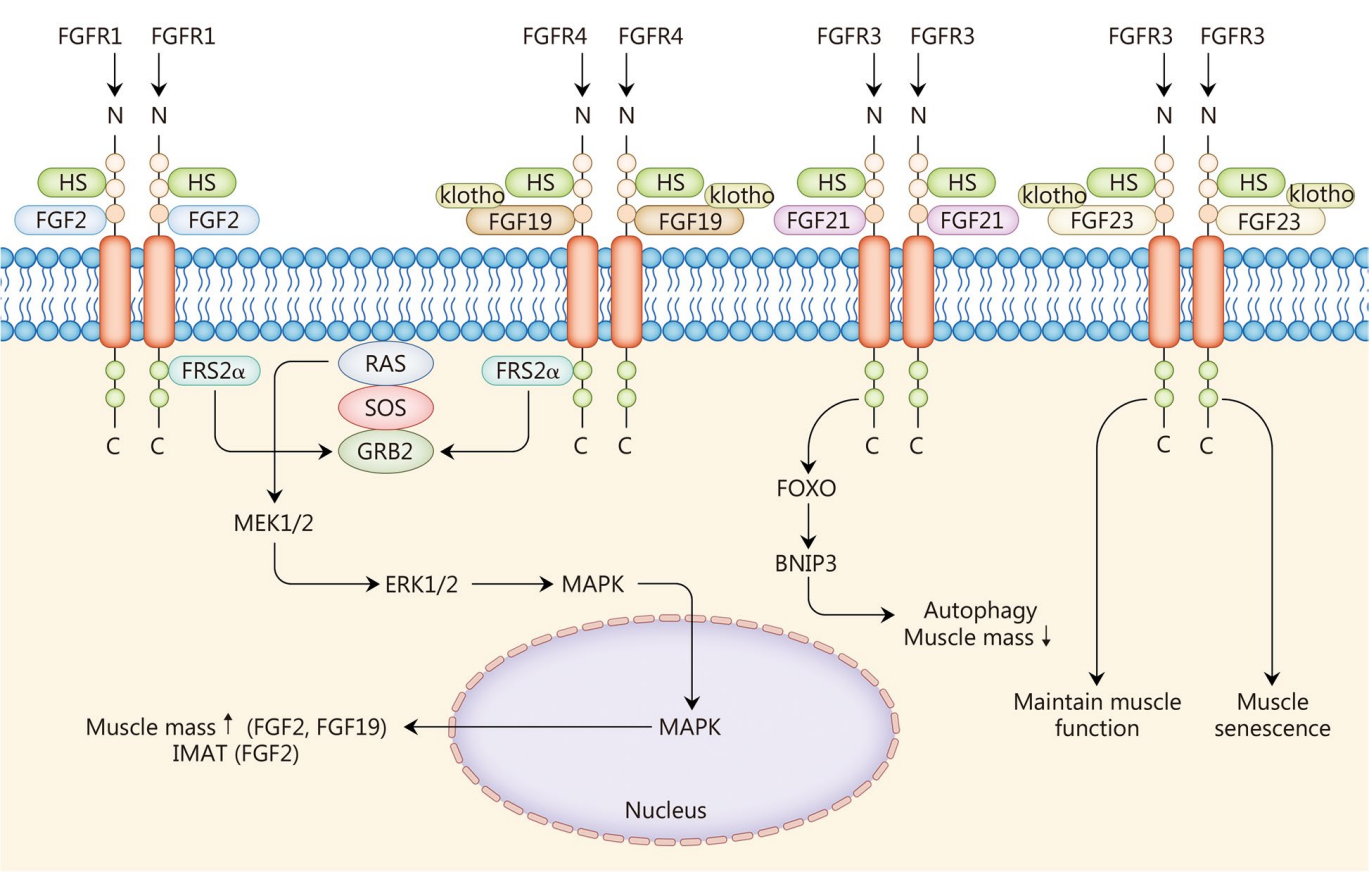
Fibroblast growth factors (FGFs) in sarcopenia. FGF2 and FGF19 increase muscle mass through the RAS-MAPK signaling pathway, and FGF2 promotes IMAT. FGF21 promotes autophagy of muscle tissue and decreases muscle mass. FGF23 accelerates the senescence of muscle tissue and maintains muscle function. IMAT intramuscular adipose tissue, FOXO forkhead box protein O, BNIP3 Bcl-2/E1B-19 kD interacting protein 3, HS heparan sulfates, FRS2α FGFR substrates 2α, SOS son of sevenless, MEK mitogen-activated extracellular, MAPK mitogen-activated protein kinase, ERK extracellular signal-regulated kinase

Sarcopenia is an age-associated degeneration condition that significantly compromises the overall well-being and health of older individuals, leading to a decline in their quality of life. The primary manifestation of sarcopenia is a gradual reduction in both muscle mass and function, which subsequently increases the risk of physical disability, mortality, and other detrimental outcomes [188]. Due to variations among ethnic groups, countries, and regions, the global prevalence of sarcopenia ranges from 9.9% to 40.0% [189], imposing a substantial burden on medical services, healthcare systems, and elderly care facilities. Muscle growth is regulated by various factors; among them are several members of the FGF family such as FGF2 that play crucial roles in modulating muscle growth and metabolism [151–159] (Fig. 5, Table 2).

### FGF2

In vitro experiments have shown that FGF2 accelerates muscle loss caused by malnutrition and promotes muscle regeneration [152]. FGF2 activates the downstream RAS-MAPK signaling pathway through FGFR1, thereby mitigating skeletal muscle loss and damage. The expression of FGF2 gradually declines with age, leading to diminished muscle growth [190]. However, FGF2 plays diverse roles in skeletal muscle. Elevated levels of FGF2 expression promote muscle growth and increase the generation of intramuscular adipose tissue (IMAT). FGF2 also regulates downstream signaling through MEK1/2 and facilitates IMAT generation [153]. Given that fat infiltration affects skeletal muscle function, it is crucial to regulate downstream molecular pathways and minimize IMAT generation when developing FGF2-related molecular drugs for regulating muscle tissue growth.

### FGF19

A cross-sectional study demonstrated a significant decrease in serum FGF19 levels and a significant increase in serum FGF21 levels among elderly individuals with sarcopenia compared to those without sarcopenia [156]. Animal and in vitro experiments have revealed that FGF19 plays a role in regulating skeletal muscle function and mitigating muscle atrophy [191]. FGF19 stimulates and activates the ERK1/2 signaling pathway as well as ribosomal protein S6 kinase 1 (S6K1), which is a key mammalian target of rapamycin (mTOR)-dependent regulator promoting muscle cell growth [154]. Activation of FXR-FGF15/19 signaling in the ileum has been found to reduce muscle atrophy in the elderly [192]. The signal transduction in this pathway may be influenced by the composition of the host intestinal flora [193]. In addition to enhancing skeletal muscle quality and function, FGF19 also safeguards against obesity-induced muscle atrophy and steatosis [155]. The findings from these diverse experiments have demonstrated that FGF19 alleviates age-related, disease-induced, or abnormal metabolic state-induced muscle atrophy while modulating carbohydrate and lipid metabolism as well as myocyte proliferation through diverse intracellular and extracellular signaling pathways.

### FGF21

The therapeutic application of FGF21 extends to carbohydrate and fat metabolic disorders, including type 2 diabetes mellitus (T2DM) and obesity [170]. An increasing body of research has explored the relationship between FGF21 expression, aging, and muscle tissue status. Elderly individuals with elevated serum levels exhibit reduced muscle strength and quality, as well as an increased susceptibility to sarcopenia [157]. Conversely, certain studies have indicated that high levels of FGF21 are associated with muscle strength rather than muscle mass [194]. Due to variations in ethnic representation across existing controlled trials and cohort studies, the reported findings may differ. Overall, heightened serum levels of FGF21 have consistently been linked to a decline in muscle strength and muscle atrophy among elderly individuals.

However, some studies have proposed a beneficial role for FGF21 in maintaining body homeostasis [195, 196]. Autophagy-deficient mouse muscle cells or those with mitochondrial dysfunction can reduce fat production by secreting FGF21, thereby preventing obesity and insulin resistance [197]. Elevated expression of FGF21 expression enhances liver sensitivity to insulin, promotes lipid oxidation and ketone production, and inhibits cell growth and bone loss, ultimately extending the lifespan of mice [198]. In cases of muscle tissue damage or metabolic abnormalities under stress conditions, myocytes secrete increased levels of FGF21 into the bloodstream to improve whole-body metabolism [199]. Nevertheless, it is worth noting that FGF21 is also considered a marker of mitochondrial myopathy and aging [200, 201].

FGF21 is highly expressed in muscle tissue during periods of fasting, metabolic disorders, and mitochondrial myopathy. During low nutritional supply conditions like fasting, FGF21 is abundantly produced and activates the mitochondrial autophagy protein BNIP3, which induces muscle mitochondrial autophagy. Elevated levels of FGF21 expression contribute to muscle atrophy and a decline in muscle function [202]. Additionally, FGF21 collaborates with other proteins such as optic tropism 1 protein that are associated with stress and aging-related signals. The activation of FGF21 occurs through the downstream FOXO signaling pathway, leading to protein decomposition and subsequent loss of muscle mass. In cases of inflammation and aging, there is an interaction between FGF21 and the highly expressed inflammatory factors IL-6 and IL-1α, resulting in a synergistic effect on lipid metabolism and muscle homeostasis interaction [203].

Current FGF21-related drugs and therapies primarily target obesity and dysregulated glucose and lipid metabolism, including conditions such as liver steatosis, T2DM, and viral hepatitis [204, 205]. As a myokine secreted by muscle tissue, FGF21 is involved in the regulation of glucose and lipid metabolism. In response to mitochondrial and endoplasmic reticulum stress accompanied by excessive secretion of inflammatory factors, the expression of FGF21 is upregulated in muscle tissue, making it a potential biomarker for disease status assessment. Although muscle tissue secretes FGF21 to regulate overall metabolic homeostasis, its activity can also be influenced by signals from other tissues or proteins. Serving as an important regulator of metabolism in muscles, FGF21 acts protectively during periods of stress-induced autophagy in muscle tissue which may lead to muscular atrophy and functional decline. Future research on drug development and treatment strategies should aim to elucidate the specific mechanisms through which FGF21 operates within muscle tissue to preserve both musculoskeletal integrity while mitigating systemic stress.

### FGF23

FGF23 is a hormone-like protein secreted by osteocytes, which plays a crucial role in regulating calcium and phosphorus metabolism through its binding to klotho and activation of FGFR [206]. The absence of klotho in mice leads to elevated phosphate levels and premature senility symptoms, indicating a close relationship between klotho and aging [207]. A cross-sectional study showed a positive correlation between high FGF23 levels and frailty among elderly individuals [158]. Furthermore, oxidative stress-induced senescence in skeletal muscle mesenchymal stem cells can be induced by FGF23 [159]. Conversely, FGF23 inhibits reactive oxygen species (ROS) production, thereby enhancing skeletal muscle motor function [208]. Although the specific role of FGF23 in muscle tissue remains largely unexplored, it is believed to promote muscle cell aging, increase muscle mass, and improve muscle function. However, further research is needed to investigate the combined effects of FGF23 with FGFR as well as the intricate interplay between klotho and FGFR signaling pathways. It should be noted that excessive activation of FGF23 may have detrimental effects on the body such as hypophosphatemia and bone loss. Nevertheless, the impact of FGF23 on the biochemical and metabolic status of muscle tissue along with its underlying mechanisms still requires elucidation.

Other FGFs also regulate the growth of muscle tissue. For example, FGF6 enhances muscle tissue regeneration through ERK1/2 signaling [209] and facilitates the repair of muscle damage after exercise. FGF9 and its subfamily members, FGF16 and FGF20, inhibit myogenic differentiation while promoting myoblast proliferation. The inhibition of differentiation and promotion of proliferation are independent functions [210]. Numerous members within the FGF family directly impact the regeneration and differentiation processes of muscle stem cells, myoblasts, and muscle tissue through endocrine or metabolic regulation, thus promoting the onset of sarcopenia. Muscle tissue can secrete certain FGFs such as FGF21, whereas bone tissue can also secrete FGFs to influence muscle anabolism. Further investigation is required to understand how signal crosstalk between skeletal muscle and bone can be balanced, as well as how FGF signaling can be regulated in various tissues and organs throughout the body. Future research should focus on elucidating specific mechanisms underlying FGF signaling and related drug development.

## Conclusions and perspective

In this review, we have shown the crucial role of the FGF-FGFR signaling pathway in the pathogenesis and progression of orthopedic degenerative diseases. In the pathology of OA, there is an upregulation of FGF1, FGF2, FGF8, and FGF23 along with a downregulation of FGF9 and FGF18, leading to cartilage degeneration, apoptosis, and alterations in matrix composition as well as calcium and phosphorus levels. Dysregulated FGFR signaling also contributes significantly to cartilage-related disorders such as OA by mediating downstream pathways through increased FGFR1 expression and decreased FGFR3 expression. Similarly, IVDD is characterized by disrupted function of FGF2, reduced expression of FGF18, and imbalanced proportions of FGFRs. Mechanical stress induces upregulation of FGF9 which results in ligamentum flavum hypertrophy and spinal stenosis [211, 212], thereby exacerbating pain symptoms. OP and sarcopenia involve the regulation of bone and muscle metabolism by various members including endocrine FGFs (FGF19 and FGF23). The expression of FGF2 is downregulated, resulting in weakened osteoblast activity and reduced bone tissue density. FGF19 exerts its effects on bone through metabolic pathways by regulating glucose and lipid metabolism, activating osteoblasts, inhibiting osteoclasts, and maintaining BMD. Therefore, a decrease in FGF19 expression may be associated with OP. In OP, there is a high expression of FGF23 which acts via the endocrine system to affect PTH levels and reduce 1,25(OH)2D3 synthesis, leading to reduced BMD. It also directly inhibits osteoblast activity and reduces bone mineral synthesis. In sarcopenia, FGF2 induces IMAT while promoting muscle growth and repair. The expression of FGF19 is decreased in sarcopenia where it regulates the metabolism of glucose and lipids in muscle tissue to increase muscle mass. FGF21 is an aging marker that is upregulated in sarcopenia causing muscle decomposition and bone loss while also regulating systemic glucose and lipid metabolism for maintaining body stability. Lastly, high expression of FGF23 can induce aging in muscle tissues with a positive correlation observed between high levels of FGF23 expression and frailty in elderly individuals.

Thus, the activity of the FGF family in bone, cartilage, and muscle plays an essential role in regulating tissue regeneration, differentiation, and metabolism, as well as the occurrence and progression of associated age-related diseases. Clinical trials have also been conducted on FGF-related drugs. Despite being promising candidates for treating degenerative bone diseases, there are still important issues that researchers need to address regarding the FGF family. For example, while progress has been made in understanding the downstream pathways regulated by FGF and FGFR, a comprehensive analysis of the FGF regulatory network and its involvement in the pathological mechanisms of degenerative diseases is still lacking. Additionally, most studies have relied on mouse or rat disease models t which may not accurately reflect human conditions. In future research endeavors, constructing animal disease models using pigs, sheep, or even monkeys would provide better insights into the functions of the FGF family. It is worth mentioning that clinical drug trials for FGF18 and FGF2 are currently underway; however, their non-selective activation of FGFR1 and FGFR3 raises uncertainties about their therapeutic efficacy. Furthermore, the concentration-dependent biphasic effects of FGFs make it challenging to determine the specific treatment timings and dosages for drugs targeting these proteins. Additionally, current FGFR inhibitors mostly function as broad-spectrum inhibitors that carry risks such as hyperphosphatemia owing to the inhibition of FGFR1. Therefore, the development of targeted and selective FGFR inhibitors remains a key focus of future research in this field.

Therefore, in future investigations into the unique mechanism of action of FGF in orthopedic degenerative diseases and the identification of therapeutic- and prevention-specific targets, it is crucial to also consider the impact of FGF on systemic metabolism and other tissues and organs. This is because FGF plays a vital role in maintaining the homeostasis of bile acids, glucose, lipids, energy, and minerals through modulation of the functional interactions between multiple organs and driving multiple metabolic axes [213]. Consequently, combining FGF with the piezoelectric hydrogel developed by Wu et al. [214] for precise and sustained drug delivery at the lesion site holds promising potential for future therapeutic programs.

In conclusion, the FGF signaling pathway plays a pivotal role in the study of orthopedic degenerative diseases. A deeper comprehension of the regulatory function of the FGF signaling pathway in the pathogenesis of orthopedic degenerative diseases will establish a foundation for the development of new treatment strategies. Exploring inhibition of the FGF signaling pathway as well as its crosstalk with other signaling pathways may generate innovative concepts for personalized medicine, enabling tailored treatment options based on patient-specific signaling pathways. The most promising research areas include: 1) elucidating the intricate fine-tuning mechanism of the FGF signaling pathway in orthopedic degenerative diseases, 2) investigating the interplay between the FGF signaling pathway and other signaling pathways to provide novel precision therapy approaches, and 3) expanding the diversity of therapeutic modalities targeting the FGF signaling pathway in the treatment of orthopedic degenerative diseases.

## Data Availability

Not applicable.

## Abbreviations

ADAMTS-5: A disintegrin and metalloproteinase thrombospondin motifs-5
AF: Annulus fibrosus
AKT: Protein kinase B
BMD: Bone mineral density
BMP: Bone morphogenetic protein
CKD: Chronic kidney disease
Col-1: Type I collagen
D: Domains
ECM: Extracellular matrix
ERK: Extracellular signal-regulated kinase
FGF: Fibroblast growth factor
FGFR: Fibroblast growth factor receptor
FRS: FGFR substrates
GSK3β: Glycogen synthase kinase-3β
IGF: Insulin-like growth factor
IL: Interleukin
IMAT: Intramuscular adipose tissue
IVDD: Intervertebral disc degeneration
IVDs: Intervertebral discs
JNK: C-Jun N-terminal kinase
MAPK: Mitogen-activated protein kinase
MEK: mitogen-activated extracellular signal-regulated kinase
MMP: Matrix metalloproteinase
NP: Nucleus pulposus
OA: Osteoarthritis
OP: Osteoporosis
PG: Proteoglycan
PI3K: Phosphatidylinositol-3-kinase
PLC: Phospholipase C
PTH: Parathyroid hormone
rAAV: Recombinant adeno-associated virus
rhFGF18: Recombinant human FGF18
RUNX2: Runt-related transcription factor 2
STAT: Signal transduction and activator of transcription
SOX9: SRY-related HMG-box gene 9
TKR: Total knee replacement
TNF-α: Tumor necrosis factor-α
XLH: X-linked hypophosphatemia

## References

[1] Yun YR, Won JE, Jeon E, Lee S, Kang W, Jo H (2010). Fibroblast growth factors: biology, function, and application for tissue regeneration. J Tissue Eng.

[2] Potthoff MJ, Kliewer SA, Mangelsdorf DJ (2012). Endocrine fibroblast growth factors 15/19 and 21: from feast to famine. Genes Dev.

[3] Bailey KN, Alliston T (2022). At the crux of joint crosstalk: TGF-β signaling in the synovial joint. Curr Rheumatol Rep..

[4] Xie Y, Zinkle A, Chen L, Mohammadi M (2020). Fibroblast growth factor signalling in osteoarthritis and cartilage repair. Nat Rev Rheumatol.

[5] Oh WT, Yang YS, Xie J, Ma H, Kim JM, Park KH (2023). WNT-modulating gene silencers as a gene therapy for osteoporosis, bone fracture, and critical-sized bone defects. Mol Ther.

[6] Xiong Y, Mi BB, Lin Z, Hu YQ, Yu L, Zha KK (2022). The role of the immune microenvironment in bone, cartilage, and soft tissue regeneration: from mechanism to therapeutic opportunity. Mil Med Res.

[7] Kubota S, Aoyama E, Takigawa M, Nishida T (2022). Fibroblast growth factors and cellular communication network factors: intimate interplay by the founding members in cartilage. Int J Mol Sci.

[8] Sun H, Sherrier M, Li H (2021). Skeletal muscle and bone - emerging targets of fibroblast growth factor-21. Front Physiol.

[9] Kisand K, Tamm AE, Lintrop M, Tamm AO (2018). New insights into the natural course of knee osteoarthritis: early regulation of cytokines and growth factors, with emphasis on sex-dependent angiogenesis and tissue remodeling A pilot study. Osteoarthr Cartil.

[10] Xu J, Huang Z, Wang W, Tan X, Li H, Zhang Y (2018). FGF8 signaling alters the osteogenic cell fate in the hard palate. J Dent Res.

[11] Lu S, Lin C-W (2021). Lentivirus-mediated transfer of gene encoding fibroblast growth factor-18 inhibits intervertebral disc degeneration. Exp Ther Med.

[12] Ornitz DM, Itoh N (2015). The fibroblast growth factor signaling pathway. Wiley Interdiscip Rev Dev Biol.

[13] Itoh N, Ornitz DM (2004). Evolution of the Fgf and Fgfr gene families. Trends Genet.

[14] Lee PL, Johnson DE, Cousens LS, Fried VA, Williams LT (1989). Purification and complementary DNA cloning of a receptor for basic fibroblast growth factor. Science.

[15] Beenken A, Mohammadi M (2009). The FGF family: biology, pathophysiology and therapy. Nat Rev Drug Discov.

[16] Zhang X, Ibrahimi OA, Olsen SK, Umemori H, Mohammadi M, Ornitz DM (2006). Receptor specificity of the fibroblast growth factor family. The complete mammalian FGF family. J Biol Chem.

[17] Hu MC, Shiizaki K, Kuro-o M, Moe OW (2013). Fibroblast growth factor 23 and Klotho: physiology and pathophysiology of an endocrine network of mineral metabolism. Annu Rev Physiol.

[18] Mohammadi M, Olsen SK, Ibrahimi OA (2005). Structural basis for fibroblast growth factor receptor activation. Cytokine Growth Factor Rev.

[19] Yayon A, Klagsbrun M, Esko JD, Leder P, Ornitz DM (1991). Cell surface, heparin-like molecules are required for binding of basic fibroblast growth factor to its high affinity receptor. Cell.

[20] Mohammadi M, Dikic I, Sorokin A, Burgess WH, Jaye M, Schlessinger J (1996). Identification of six novel autophosphorylation sites on fibroblast growth factor receptor 1 and elucidation of their importance in receptor activation and signal transduction. Mol Cell Biol.

[21] Mohammadi M, Honegger AM, Rotin D, Fischer R, Bellot F, Li W (1991). A tyrosine-phosphorylated carboxy-terminal peptide of the fibroblast growth factor receptor (Flg) is a binding site for the SH2 domain of phospholipase C-gamma 1. Mol Cell Biol.

[22] Haugsten EM, Sørensen V, Brech A, Olsnes S, Wesche J (2005). Different intracellular trafficking of FGF1 endocytosed by the four homologous FGF receptors. J Cell Sci.

[23] Johnson DE, Williams LT (1993). Structural and functional diversity in the FGF receptor multigene family. Adv Cancer Res.

[24] Ornitz DM (2000). FGFs, heparan sulfate and FGFRs: complex interactions essential for development. Bioessays.

[25] Raffioni S, Thomas D, Foehr ED, Thompson LM, Bradshaw RA (1999). Comparison of the intracellular signaling responses by three chimeric fibroblast growth factor receptors in PC12 cells. Proc Natl Acad Sci U S A.

[26] Dailey L, Ambrosetti D, Mansukhani A, Basilico C (2005). Mechanisms underlying differential responses to FGF signaling. Cytokine Growth Factor Rev.

[27] Lamothe B, Yamada M, Schaeper U, Birchmeier W, Lax I, Schlessinger J (2004). The docking protein Gab1 is an essential component of an indirect mechanism for fibroblast growth factor stimulation of the phosphatidylinositol 3-kinase/Akt antiapoptotic pathway. Mol Cell Biol.

[28] Su WC, Kitagawa M, Xue N, Xie B, Garofalo S, Cho J (1997). Activation of Stat1 by mutant fibroblast growth-factor receptor in thanatophoric dysplasia type II dwarfism. Nature.

[29] Sahni M, Ambrosetti DC, Mansukhani A, Gertner R, Levy D, Basilico C (1999). FGF signaling inhibits chondrocyte proliferation and regulates bone development through the STAT-1 pathway. Genes Dev.

[30] Heath C, Cross NCP (2004). Critical role of STAT5 activation in transformation mediated by ZNF198-FGFR1. J Biol Chem.

[31] Teven CM, Farina EM, Rivas J, Reid RR (2014). Fibroblast growth factor (FGF) signaling in development and skeletal diseases. Genes Dis.

[32] Dorey K, Amaya E (2010). FGF signalling: diverse roles during early vertebrate embryogenesis. Development.

[33] Wong A, Lamothe B, Lee A, Schlessinger J, Lax I (2002). FRS2 alpha attenuates FGF receptor signaling by Grb2-mediated recruitment of the ubiquitin ligase Cbl. Proc Natl Acad Sci U S A.

[34] Harris VK, Kagan BL, Ray R, Coticchia CM, Liaudet-Coopman ED, Wellstein A (2001). Serum induction of the fibroblast growth factor-binding protein (FGF-BP) is mediated through ERK and p38 MAP kinase activation and C/EBP-regulated transcription. Oncogene.

[35] Cinque L, De Leonibus C, Iavazzo M, Krahmer N, Intartaglia D, Salierno FG (2020). MiT/TFE factors control ER-phagy via transcriptional regulation of FAM134B. EMBO J.

[36] Clark JF, Soriano P (2023). FRS2-independent GRB2 interaction with FGFR2 is not required for embryonic development. Biol Open.

[37] Zakrzewska M, Opalinski L, Haugsten EM, Otlewski J, Wiedlocha A (2019). Crosstalk between p38 and Erk 1/2 in downregulation of FGF1-induced signaling. Int J Mol Sci.

[38] Li F, Cai T, Yu L, Yu G, Zhang H, Geng Y (2024). FGF-18 protects the injured spinal cord in mice by suppressing pyroptosis and promoting autophagy via the AKT-mTOR-TRPML1 axis. Mol Neurobiol.

[39] Jin M, Du X, Chen L (2012). Cross-talk between FGF and other cytokine signalling pathways during endochondral bone development. Cell Biol Int.

[40] Peters KG, Marie J, Wilson E, Ives HE, Escobedo J, Del Rosario M (1992). Point mutation of an FGF receptor abolishes phosphatidylinositol turnover and Ca2+ flux but not mitogenesis. Nature.

[41] Kolkova K, Novitskaya V, Pedersen N, Berezin V, Bock E (2000). Neural cell adhesion molecule-stimulated neurite outgrowth depends on activation of protein kinase C and the Ras-mitogen-activated protein kinase pathway. J Neurosci.

[42] Dudka AA, Sweet SMM, Heath JK (2010). Signal transducers and activators of transcription-3 binding to the fibroblast growth factor receptor is activated by receptor amplification. Cancer Res.

[43] Kolasinski SL, Neogi T, Hochberg MC, Oatis C, Guyatt G, Block J (2020). 2019 American college of rheumatology/arthritis foundation guideline for the management of osteoarthritis of the hand, hip, and knee. Arthritis Care Res (Hoboken).

[44] Ulrich-Vinther M, Maloney MD, Schwarz EM, Rosier R, O'Keefe RJ (2003). Articular cartilage biology. J Am Acad Orthop Surg.

[45] Chen TM, Chen YH, Sun HS, Tsai SJ (2019). Fibroblast growth factors: potential novel targets for regenerative therapy of osteoarthritis. Chin J Physiol.

[46] Silverwood V, Blagojevic-Bucknall M, Jinks C, Jordan JL, Protheroe J, Jordan KP (2015). Current evidence on risk factors for knee osteoarthritis in older adults: a systematic review and meta-analysis. Osteoarthr Cartil.

[47] Christensen R, Bartels EM, Astrup A, Bliddal H (2007). Effect of weight reduction in obese patients diagnosed with knee osteoarthritis: a systematic review and meta-analysis. Ann Rheum Dis.

[48] Sanchez-Lopez E, Coras R, Torres A, Lane NE, Guma M (2022). Synovial inflammation in osteoarthritis progression. Nat Rev Rheumatol.

[49] Goldring MB, Goldring SR (2010). Articular cartilage and subchondral bone in the pathogenesis of osteoarthritis. Ann N Y Acad Sci.

[50] Wojdasiewicz P, Poniatowski ŁA, Szukiewicz D (2014). The role of inflammatory and anti-inflammatory cytokines in the pathogenesis of osteoarthritis. Mediators Inflamm.

[51] Mort JS, Billington CJ (2001). Articular cartilage and changes in arthritis: matrix degradation. Arthritis Res.

[52] Li R, Wang B, He CQ, Yang YQ, Guo H, Chen Y (2015). Upregulation of fibroblast growth factor 1 in the synovial membranes of patients with late stage osteoarthritis. Genet Mol Res.

[53] Li ZC, Xiao J, Wang G, Li MQ, Hu KZ, Ma T (2015). Fibroblast growth factor-21 concentration in serum and synovial fluid is associated with radiographic bone loss of knee osteoarthritis. Scand J Clin Lab Invest.

[54] Wu L, Leijten J, van Blitterswijk CA, Karperien M (2013). Fibroblast growth factor-1 is a mesenchymal stromal cell-secreted factor stimulating proliferation of osteoarthritic chondrocytes in co-culture. Stem Cells Dev.

[55] El-Seoudi A, Abd El Kader T, Nishida T, Eguchi T, Aoyama E, Takigawa M (2017). Catabolic effects of FGF-1 on chondrocytes and its possible role in osteoarthritis. J Cell Commun Signal.

[56] Yan D, Chen D, Cool SM, van Wijnen AJ, Mikecz K, Murphy G (2011). Fibroblast growth factor receptor 1 is principally responsible for fibroblast growth factor 2-induced catabolic activities in human articular chondrocytes. Arthritis Res Ther.

[57] Vincent T, Hermansson M, Bolton M, Wait R, Saklatvala J (2002). Basic FGF mediates an immediate response of articular cartilage to mechanical injury. Proc Natl Acad Sci U S A.

[58] Wang X, Manner PA, Horner A, Shum L, Tuan RS, Nuckolls GH (2004). Regulation of MMP-13 expression by RUNX2 and FGF2 in osteoarthritic cartilage. Osteoarthr Cartil.

[59] Im HJ, Muddasani P, Natarajan V, Schmid TM, Block JA, Davis F (2007). Basic fibroblast growth factor stimulates matrix metalloproteinase-13 via the molecular cross-talk between the mitogen-activated protein kinases and protein kinase Cdelta pathways in human adult articular chondrocytes. J Biol Chem.

[60] Yan D, Chen D, Im HJ (2012). Fibroblast growth factor-2 promotes catabolism via FGFR1-Ras-Raf-MEK1/2-ERK1/2 axis that coordinates with the PKCdelta pathway in human articular chondrocytes. J Cell Biochem.

[61] Vincent TL (2011). Fibroblast growth factor 2: good or bad guy in the joint?. Arthritis Res Ther.

[62] Liu H, Fang Q, Wang M, Wang W, Zhang M, Zhang D (2018). FGF8 and FGFR3 are up-regulated in hypertrophic chondrocytes: association with chondrocyte death in deep zone of Kashin-Beck disease. Biochem Biophys Res Commun.

[63] Pujol JP, Loyau G (1987). Interleukin-1 and osteoarthritis. Life Sci.

[64] Zhao Y, Li Y, Qu R, Chen X, Wang W, Qiu C (2019). Cortistatin binds to TNF-α receptors and protects against osteoarthritis. EBioMedicine.

[65] Zhou S, Wang Z, Tang J, Li W, Huang J, Xu W (2016). Exogenous fibroblast growth factor 9 attenuates cartilage degradation and aggravates osteophyte formation in post-traumatic osteoarthritis. Osteoarthr Cartil.

[66] Gigout A, Guehring H, Froemel D, Meurer A, Ladel C, Reker D (2017). Sprifermin (rhFGF18) enables proliferation of chondrocytes producing a hyaline cartilage matrix. Osteoarthr Cartil.

[67] Müller S, Lindemann S, Gigout A (2020). Effects of Sprifermin, IGF1, IGF2, BMP7, or CNP on bovine chondrocytes in monolayer and 3D Culture. J Orthop Res.

[68] Eckstein F, Hochberg MC, Guehring H, Moreau F, Ona V, Bihlet AR (2021). Long-term structural and symptomatic effects of intra-articular sprifermin in patients with knee osteoarthritis: 5-year results from the FORWARD study. Ann Rheum Dis.

[69] Beck-Nielsen SS, Mughal Z, Haffner D, Nilsson O, Levtchenko E, Ariceta G (2019). FGF23 and its role in X-linked hypophosphatemia-related morbidity. Orphanet J Rare Dis.

[70] Orfanidou T, Iliopoulos D, Malizos KN, Tsezou A (2009). Involvement of SOX-9 and FGF-23 in RUNX-2 regulation in osteoarthritic chondrocytes. J Cell Mol Med.

[71] Nishida T, Kubota S (2020). Roles of CCN2 as a mechano-sensing regulator of chondrocyte differentiation. Jpn Dent Sci Rev.

[72] Chapman JR, Katsara O, Ruoff R, Morgenstern D, Nayak S, Basilico C (2017). Phosphoproteomics of fibroblast growth factor 1 (FGF1) signaling in chondrocytes: identifying the signature of inhibitory response. Mol Cell Proteomics.

[73] Yamamoto T, Wakitani S, Imoto K, Hattori T, Nakaya H, Saito M (2004). Fibroblast growth factor-2 promotes the repair of partial thickness defects of articular cartilage in immature rabbits but not in mature rabbits. Osteoarthr Cartil.

[74] Chong KW, Chanalaris A, Burleigh A, Jin H, Watt FE, Saklatvala J (2013). Fibroblast growth factor 2 drives changes in gene expression following injury to murine cartilage in vitro and in vivo. Arthritis Rheum.

[75] Burleigh A, Chanalaris A, Gardiner MD, Driscoll C, Boruc O, Saklatvala J (2012). Joint immobilization prevents murine osteoarthritis and reveals the highly mechanosensitive nature of protease expression in vivo. Arthritis Rheum.

[76] Blunt AG, Lawshé A, Cunningham ML, Seto ML, Ornitz DM, MacArthur CA (1997). Overlapping expression and redundant activation of mesenchymal fibroblast growth factor (FGF) receptors by alternatively spliced FGF-8 ligands. J Biol Chem.

[77] Uchii M, Tamura T, Suda T, Kakuni M, Tanaka A, Miki I (2008). Role of fibroblast growth factor 8 (FGF8) in animal models of osteoarthritis. Arthritis Res Ther.

[78] Shakibaei M, Schulze-Tanzil G, John T, Mobasheri A (2005). Curcumin protects human chondrocytes from IL-l1beta-induced inhibition of collagen type II and beta1-integrin expression and activation of caspase-3: an immunomorphological study. Ann Anat.

[79] Saklatvala J (1986). Tumour necrosis factor alpha stimulates resorption and inhibits synthesis of proteoglycan in cartilage. Nature.

[80] Grunke M, Schulze-Koops H (2006). Successful treatment of inflammatory knee osteoarthritis with tumour necrosis factor blockade. Ann Rheum Dis.

[81] Chevalier X, Giraudeau B, Conrozier T, Marliere J, Kiefer P, Goupille P (2005). Safety study of intraarticular injection of interleukin 1 receptor antagonist in patients with painful knee osteoarthritis: a multicenter study. J Rheumatol.

[82] Hecht D, Zimmerman N, Bedford M, Avivi A, Yayon A (1995). Identification of fibroblast growth factor 9 (FGF9) as a high affinity, heparin dependent ligand for FGF receptors 3 and 2 but not for FGF receptors 1 and 4. Growth Factors.

[83] Wang L, Roth T, Abbott M, Ho L, Wattanachanya L, Nissenson RA (2017). Osteoblast-derived FGF9 regulates skeletal homeostasis. Bone.

[84] Pan YN, Jia C, Yu JP, Wu ZW, Xu GC, Huang YX (2023). Fibroblast growth factor 9 reduces TBHP-induced oxidative stress in chondrocytes and diminishes mouse osteoarthritis by activating ERK/Nrf2 signaling pathway. Int Immunopharmacol.

[85] Correa D, Somoza RA, Lin P, Greenberg S, Rom E, Duesler L (2015). Sequential exposure to fibroblast growth factors (FGF) 2, 9 and 18 enhances hMSC chondrogenic differentiation. Osteoarthr Cartil.

[86] Yuan WH, Xie QQ, Wang KP, Shen W, Feng XF, Liu Z (2021). Screening of osteoarthritis diagnostic markers based on immune-related genes and immune infiltration. Sci Rep.

[87] Davidson D, Blanc A, Filion D, Wang H, Plut P, Pfeffer G (2005). Fibroblast growth factor (FGF) 18 signals through FGF receptor 3 to promote chondrogenesis. J Biol Chem.

[88] Liu Z, Lavine KJ, Hung IH, Ornitz DM (2007). FGF18 is required for early chondrocyte proliferation, hypertrophy and vascular invasion of the growth plate. Dev Biol.

[89] Moore EE, Bendele AM, Thompson DL, Littau A, Waggie KS, Reardon B (2005). Fibroblast growth factor-18 stimulates chondrogenesis and cartilage repair in a rat model of injury-induced osteoarthritis. Osteoarthr Cartil.

[90] Reinhold MI, Abe M, Kapadia RM, Liao Z, Naski MC (2004). FGF18 represses noggin expression and is induced by calcineurin. J Biol Chem.

[91] Li X, An HS, Ellman M, Phillips F, Thonar EJ, Park DK (2008). Action of fibroblast growth factor-2 on the intervertebral disc. Arthritis Res Ther.

[92] Zeng N, Chen XY, Yan ZP, Li JT, Liao T, Ni GX (2021). Efficacy and safety of sprifermin injection for knee osteoarthritis treatment: a meta-analysis. Arthritis Res Ther.

[93] Guehring H, Moreau F, Daelken B, Ladel C, Guenther O, Bihlet AR (2021). The effects of sprifermin on symptoms and structure in a subgroup at risk of progression in the FORWARD knee osteoarthritis trial. Semin Arthritis Rheum.

[94] Power J, Hernandez P, Guehring H, Getgood A, Henson F (2014). Intra-articular injection of rhFGF-18 improves the healing in microfracture treated chondral defects in an ovine model. J Orthop Res.

[95] Barr L, Getgood A, Guehring H, Rushton N, Henson FM (2014). The effect of recombinant human fibroblast growth factor-18 on articular cartilage following single impact load. J Orthop Res.

[96] Reker D, Siebuhr AS, Thudium CS, Gantzel T, Ladel C, Michaelis M (2020). Sprifermin (rhFGF18) versus vehicle induces a biphasic process of extracellular matrix remodeling in human knee OA articular cartilage ex vivo. Sci Rep.

[97] Lohmander LS, Hellot S, Dreher D, Krantz EF, Kruger DS, Guermazi A (2014). Intraarticular sprifermin (recombinant human fibroblast growth factor 18) in knee osteoarthritis: a randomized, double-blind, placebo-controlled trial. Arthritis Rheumatol.

[98] Ewendt F, Föller M (2019). p38MAPK controls fibroblast growth factor 23 (FGF23) synthesis in UMR106-osteoblast-like cells and in IDG-SW3 osteocytes. J Endocrinol Invest.

[99] BärBar L, Hase P, FöllerFoller M (2019). PKC regulates the production of fibroblast growth factor 23 (FGF23). PLoS One.

[100] Meo Burt P, Xiao L, Hurley MM (2018). FGF23 regulates Wnt/beta-catenin signaling-mediated osteoarthritis in mice overexpressing high-molecular-weight FGF2. Endocrinology.

[101] Julien A, Perrin S, Duchamp de Lageneste O, Carvalho C, Bensidhoum M, Legeai-Mallet L (2020). FGFR3 in periosteal cells drives cartilage-to-bone transformation in bone repair. Stem Cell Rep.

[102] Nagai H, Aoki M (2002). Inhibition of growth plate angiogenesis and endochondral ossification with diminished expression of MMP-13 in hypertrophic chondrocytes in FGF-2-treated rats. J Bone Miner Metab.

[103] Murakami S, Kan M, McKeehan WL, de Crombrugghe B (2000). Up-regulation of the chondrogenic Sox9 gene by fibroblast growth factors is mediated by the mitogen-activated protein kinase pathway. Proc Natl Acad Sci U S A.

[104] Harada M, Akita K (2020). Mouse fibroblast growth factor 9 N143T mutation leads to wide chondrogenic condensation of long bones. Histochem Cell Biol.

[105] Hagan AS, Boylan M, Smith C, Perez-Santamarina E, Kowalska K, Hung IH (2019). Generation and validation of novel conditional flox and inducible Cre alleles targeting fibroblast growth factor 18 (Fgf18). Dev Dyn.

[106] Mori Y, Saito T, Chang SH, Kobayashi H, Ladel CH, Guehring H (2014). Identification of fibroblast growth factor-18 as a molecule to protect adult articular cartilage by gene expression profiling. J Biol Chem.

[107] Liu Z, Xu J, Colvin JS, Ornitz DM (2002). Coordination of chondrogenesis and osteogenesis by fibroblast growth factor 18. Gene Dev.

[108] Hochberg MC, Guermazi A, Guehring H, Aydemir A, Wax S, Fleuranceau-Morel P (2019). Effect of intra-articular sprifermin vs placebo on femorotibial joint cartilage thickness in patients with osteoarthritis: the FORWARD randomized clinical trial. JAMA.

[109] Zhai F, Song N, Ma J, Gong W, Tian H, Li X (2017). FGF18 inhibits MC3T3-E1 cell osteogenic differentiation via the ERK signaling pathway. Mol Med Rep.

[110] Imamura K, Tachi K, Takayama T, Shohara R, Kasai H, Dai J (2018). Released fibroblast growth factor18 from a collagen membrane induces osteoblastic activity involved with downregulation of miR-133a and miR-135a. J Biomater Appl.

[111] Cinque L, Forrester A, Bartolomeo R, Svelto M, Venditti R, Montefusco S (2015). FGF signalling regulates bone growth through autophagy. Nature.

[112] Li X, Ellman MB, Kroin JS, Chen D, Yan D, Mikecz K (2012). Species-specific biological effects of FGF-2 in articular cartilage: implication for distinct roles within the FGF receptor family. J Cell Biochem.

[113] Ellman MB, Yan D, Ahmadinia K, Chen D, An HS, Im HJ (2013). Fibroblast growth factor control of cartilage homeostasis. J Cell Biochem.

[114] Yi L, Lan G, Ju Y, Yin X, Zhang P, Xu Y (2021). Blockade of Fgfr1 with PD166866 protects cartilage from the catabolic effects induced by interleukin-1beta: a genome-wide expression profiles analysis. Cartilage.

[115] Tan Q, Chen B, Wang Q, Xu W, Wang Y, Lin Z (2018). A novel FGFR1-binding peptide attenuates the degeneration of articular cartilage in adult mice. Osteoarthr Cartil.

[116] Weng T, Yi L, Huang J, Luo F, Wen X, Du X (2012). Genetic inhibition of fibroblast growth factor receptor 1 in knee cartilage attenuates the degeneration of articular cartilage in adult mice. Arthritis Rheum.

[117] Narayana J, Horton WA (2015). FGFR3 biology and skeletal disease. Connect Tissue Res.

[118] Valverde-Franco G, Liu H, Davidson D, Chai S, Valderrama-Carvajal H, Goltzman D (2004). Defective bone mineralization and osteopenia in young adult FGFR3-/- mice. Hum Mol Genet.

[119] Tang J, Su N, Zhou S, Xie Y, Huang J, Wen X (2016). Fibroblast growth factor receptor 3 inhibits osteoarthritis progression in the knee joints of adult mice. Arthritis Rheumatol.

[120] Krejci P, Salazar L, Kashiwada TA, Chlebova K, Salasova A, Thompson LM (2008). Analysis of STAT1 activation by six FGFR3 mutants associated with skeletal dysplasia undermines dominant role of STAT1 in FGFR3 signaling in cartilage. PLoS One.

[121] Legeai-Mallet L, Benoist-Lasselin C, Munnich A, Bonaventure J (2004). Overexpression of FGFR3, Stat1, Stat5 and p21Cip1 correlates with phenotypic severity and defective chondrocyte differentiation in FGFR3-related chondrodysplasias. Bone.

[122] Komori T (2020). Molecular mechanism of Runx2-dependent bone development. Mol Cells.

[123] Chia SL, Sawaji Y, Burleigh A, McLean C, Inglis J, Saklatvala J (2009). Fibroblast growth factor 2 is an intrinsic chondroprotective agent that suppresses ADAMTS-5 and delays cartilage degradation in murine osteoarthritis. Arthritis Rheum.

[124] Brett A, Bowes MA, Conaghan PG, Ladel C, Guehring H, Moreau F (2020). Automated MRI assessment confirms cartilage thickness modification in patients with knee osteoarthritis: post-hoc analysis from a phase II sprifermin study. Osteoarthr Cartil.

[125] Roemer FW, Kraines J, Aydemir A, Wax S, Hochberg MC, Crema MD (2020). Evaluating the structural effects of intra-articular sprifermin on cartilage and non-cartilaginous tissue alterations, based on sqMRI assessment over 2 years. Osteoarthr Cartil.

[126] Reker D, Kjelgaard-Petersen CF, Siebuhr AS, Michaelis M, Gigout A, Karsdal MA (2017). Sprifermin (rhFGF18) modulates extracellular matrix turnover in cartilage explants ex vivo. J Transl Med.

[127] Ladel CH, Ellsworth JL, Bendele AM, Gimona A, Riva S, Baur EV (2007). Therapeutic effects of fibroblast growth factor-18 in a rat model of established osteoarthritis. Osteoarthr Cartil.

[128] Ladel CH, Gimona A, Ellsworth JL, Bendele A, vom Baur E (2008). 73 recombinant human fibroblast growth factor 18 as therapy for osteoarthritis: a dog meniscectomy model study. Osteoarthr Cartil.

[129] Ladel CH, Capobianco R, Gimona A, Baur EV (2008). Functional and structural improvements in a dog anterior cruciate ligament model: recombinant human fibroblast growth factor 18 as therapy for osteoarthritis. Osteoarthr Cartil.

[130] Ladel CH, Barbero L, Riva S, Guehring H (2020). Tissue distribution of sprifermin (recombinant human fibroblast growth factor 18) in the rat following intravenous and intra-articular injection. Osteoarthr Cartil Open.

[131] Dahlberg LE, Aydemir A, Muurahainen N, Gühring H, Fredberg Edebo H, Krarup-Jensen N (2016). A first-in-human, double-blind, randomised, placebo-controlled, dose ascending study of intra-articular rhFGF18 (sprifermin) in patients with advanced knee osteoarthritis. Clin Exp Rheumatol.

[132] Cucchiarini M, Madry H, Ma C, Thurn T, Zurakowski D, Menger MD (2005). Improved tissue repair in articular cartilage defects in vivo by rAAV-mediated overexpression of human fibroblast growth factor 2. Mol Ther.

[133] Morscheid YP, Venkatesan JK, Schmitt G, Orth P, Zurakowski D, Speicher-mentges S (2021). rAAV-Mediated Human FGF-2 gene therapy enhances osteochondral repair in a clinically relevant large animal model over time in vivo. Am J Sports Med.

[134] Zomer Volpato F, Almodóvar J, Erickson K, Popat KC, Migliaresi C, Kipper MJ (2012). Preservation of FGF-2 bioactivity using heparin-based nanoparticles, and their delivery from electrospun chitosan fibers. Acta Biomater.

[135] Mori Y, Nakamura S, Kishimoto S, Kawakami M, Suzuki S, Matsui T (2010). Preparation and characterization of low-molecular-weight heparin/protamine nanoparticles (LMW-H/P NPs) as FGF-2 carrier. Int J Nanomed.

[136] Hirata E, Ménard-Moyon C, Venturelli E, Takita H, Watari F, Bianco A (2013). Carbon nanotubes functionalized with fibroblast growth factor accelerate proliferation of bone marrow-derived stromal cells and bone formation. Nanotechnology.

[137] Zulkifli A, Ahmad RE, Krishnan S, Kong P, Nam HY, Kamarul T (2023). The potential mechanism of hypoxia-induced tenogenic differentiation of mesenchymal stem cell for tendon regeneration. Tissue Cell.

[138] Al-Masawa ME, Wan Kamarul Zaman WS, Chua KH (2020). Biosafety evaluation of culture-expanded human chondrocytes with growth factor cocktail: a preclinical study. Sci Rep.

[139] Roberts S, Evans H, Trivedi J, Menage J (2006). Histology and pathology of the human intervertebral disc. J Bone Joint Surg Am.

[140] Sive JI, Baird P, Jeziorsk M, Watkins A, Hoyland JA, Freemont AJ (2002). Expression of chondrocyte markers by cells of normal and degenerate intervertebral discs. Mol Pathol.

[141] Vo NV, Hartman RA, Patil PR, Risbud MV, Kletsas D, Iatridis JC (2016). Molecular mechanisms of biological aging in intervertebral discs. J Orthop Res.

[142] Murakami H, Yoon ST, Attallah-Wasif ES, Tsai K-J, Fei Q, Hutton WC (2006). The expression of anabolic cytokines in intervertebral discs in age-related degeneration. Spine.

[143] Tolonen J, Grönblad M, Virri J, Seitsalo S, Rytömaa T, Karaharju E (1995). Basic fibroblast growth factor immunoreactivity in blood vessels and cells of disc herniations. Spine.

[144] Häckel S, Zolfaghar M, Du J, Hoppe S, Benneker LM, Garstka N (2019). Fibrin-hyaluronic acid hydrogel (RegenoGel) with fibroblast growth factor-18 for in vitro 3D culture of human and bovine nucleus pulposus cells. Int J Mol Sci.

[145] Novais A, Chatzopoulou E, Chaussain C, Gorin C (2021). The potential of FGF-2 in craniofacial bone tissue engineering: a review. Cells.

[146] Kir S, Beddow SA, Samuel VT, Miller P, Previs SF, Suino-Powell K (2011). FGF19 as a postprandial, insulin-independent activator of hepatic protein and glycogen synthesis. Science.

[147] Yu X, Xia Y, Jia J, Yuan G (2022). The role of fibroblast growth factor 19 subfamily in different populations suffering from osteoporosis. Front Endocrinol..

[148] Jelenik T, Dille M, Müller-Lühlhoff S, Kabra DG, Zhou Z, Binsch C (2018). FGF21 regulates insulin sensitivity following long-term chronic stress. Mol Metab.

[149] Bär L, Feger M, Fajol A, Klotz LO, Zeng S, Lang F (2018). Insulin suppresses the production of fibroblast growth factor 23 (FGF23). Proc Natl Acad Sci U S A.

[150] Murali SK, Roschger P, Zeitz U, Klaushofer K, Andrukhova O, Erben RG (2016). FGF23 regulates bone mineralization in a 1,25(OH)2 D3 and Klotho-independent manner. J Bone Miner Res.

[151] Homer-Bouthiette C, Xiao L, Hurley MM (2021). Gait disturbances and muscle dysfunction in fibroblast growth factor 2 knockout mice. Sci Rep.

[152] Lefaucheur JP, Sebille A (1995). Basic fibroblast growth factor promotes in vivo muscle regeneration in murine muscular dystrophy. Neurosci Lett.

[153] Mathes S, Fahrner A, Ghoshdastider U, Rüdiger HA, Leunig M, Wolfrum C (2021). FGF-2-dependent signaling activated in aged human skeletal muscle promotes intramuscular adipogenesis. Proc Natl Acad Sci U S A.

[154] Benoit B, Meugnier E, Castelli M, Chanon S, Vieille-Marchiset A, Durand C (2017). Fibroblast growth factor 19 regulates skeletal muscle mass and ameliorates muscle wasting in mice. Nat Med.

[155] Guo A, Li K, Tian HC, Fan Z, Chen QN, Yang YF (2021). FGF19 protects skeletal muscle against obesity-induced muscle atrophy, metabolic derangement and abnormal irisin levels via the AMPK/SIRT-1/PGC-α pathway. J Cell Mol Med.

[156] Bag Soytas R, Suzan V, Arman P, Emiroglu Gedik T, Unal D, Cengiz M (2021). Association of FGF-19 and FGF-21 levels with primary sarcopenia. Geriatr Gerontol Int.

[157] Jung HW, Park JH, Kim DA, Jang IY, Park SJ, Lee JY (2021). Association between serum FGF21 level and sarcopenia in older adults. Bone..

[158] Beben T, Ix JH, Shlipak MG, Sarnak MJ, Fried LF, Hoofnagle AN (2016). Fibroblast growth factor-23 and frailty in elderly community-dwelling individuals: the cardiovascular health study. J Am Geriatr Soc.

[159] Sato C, Iso Y, Mizukami T, Otabe K, Sasai M, Kurata M (2016). Fibroblast growth factor-23 induces cellular senescence in human mesenchymal stem cells from skeletal muscle. Biochem Biophys Res Commun.

[160] Pratsinis H, Constantinou V, Pavlakis K, Sapkas G, Kletsas D (2012). Exogenous and autocrine growth factors stimulate human intervertebral disc cell proliferation via the ERK and Akt pathways. J Orthop Res.

[161] Ellman MB, An HS, Muddasani P, Im HJ (2008). Biological impact of the fibroblast growth factor family on articular cartilage and intervertebral disc homeostasis. Gene.

[162] Friedl A, Chang Z, Tierney A, Rapraeger AC (1997). Differential binding of fibroblast growth factor-2 and -7 to basement membrane heparan sulfate: comparison of normal and abnormal human tissues. Am J Pathol.

[163] Loeser RF, Chubinskaya S, Pacione C, Im HJ (2005). Basic fibroblast growth factor inhibits the anabolic activity of insulin-like growth factor 1 and osteogenic protein 1 in adult human articular chondrocytes. Arthritis Rheum.

[164] Melrose J, Smith S, Little CB, Kitson J, Hwa SY, Ghosh P (2002). Spatial and temporal localization of transforming growth factor-beta, fibroblast growth factor-2, and osteonectin, and identification of cells expressing alpha-smooth muscle actin in the injured anulus fibrosus: implications for extracellular matrix repair. Spine (Phila Pa 1976).

[165] Compston JE, McClung MR, Leslie WD (2019). Osteoporosis. Lancet.

[166] Montero A, Okada Y, Tomita M, Ito M, Tsurukami H, Nakamura T (2000). Disruption of the fibroblast growth factor-2 gene results in decreased bone mass and bone formation. J Clin Invest.

[167] Fei Y, Hurley MM (2012). Role of fibroblast growth factor 2 and Wnt signaling in anabolic effects of parathyroid hormone on bone formation. J Cell Physiol.

[168] Coffin JD, Homer-Bouthiette C, Hurley MM (2018). Fibroblast growth factor 2 and its receptors in bone biology and disease. J Endoc Soc.

[169] Jilka RL (2007). Molecular and cellular mechanisms of the anabolic effect of intermittent PTH. Bone.

[170] Phan P, Saikia BB, Sonnaila S, Agrawal S, Alraawi Z, Kumar TKS (2021). The saga of endocrine FGFs. Cells..

[171] Zhao YX, Song YW, Zhang L, Zheng FJ, Wang XM, Zhuang XH (2020). Association between bile acid metabolism and bone mineral density in postmenopausal women. Clinics (Sao Paulo, Brazil).

[172] Guo A, Li K, Tian HC, Tao BL, Xiao Q, Jiang DM (2022). FGF19 protects against obesity-induced bone loss by promoting osteogenic differentiation. Biomed Pharmacother.

[173] Guridi M, Tintignac LA, Lin S, Kupr B, Castets P, Rüegg MA (2015). Activation of mTORC1 in skeletal muscle regulates whole-body metabolism through FGF21. Sci Signal.

[174] Kim AM, Somayaji VR, Dong JQ, Rolph TP, Weng Y, Chabot JR (2017). Once-weekly administration of a long-acting fibroblast growth factor 21 analogue modulates lipids, bone turnover markers, blood pressure and body weight differently in obese people with hypertriglyceridaemia and in non-human primates. Diabetes Obes Metab.

[175] Charoenphandhu N, Suntornsaratoon P, Krishnamra N, Sa-Nguanmoo P, Tanajak P, Wang X (2017). Fibroblast growth factor-21 restores insulin sensitivity but induces aberrant bone microstructure in obese insulin-resistant rats. J Bone Miner Metab.

[176] Wu YT, Hsu BG, Wang CH, Lin YL, Lai YH, Kuo CH (2020). Lower serum fibroblast growth factor 21 levels are associated with normal lumbar spine bone mineral density in hemodialysis patients. Int J Environ Res Public Health.

[177] Hao RH, Gao JL, Li M, Huang W, Zhu DL, Thynn HN (2018). Association between fibroblast growth factor 21 and bone mineral density in adults. Endocrine.

[178] Hu W, He J, Fu W, Wang C, Yue H, Gu J (2019). Fibroblast growth factor 21 is associated with bone mineral density, but not with bone turnover markers and fractures in chinese postmenopausal women. J Clin Densitom.

[179] Lui DTW, Lee CH, Chau VWK, Fong CHY, Yeung KMY, Lam JKY (2021). Potential role of fibroblast growth factor 21 in the deterioration of bone quality in impaired glucose tolerance. J Endocrinol Invest.

[180] Fernandes-Freitas I, Owen BM (2015). Metabolic roles of endocrine fibroblast growth factors. Curr Opin Pharmacol.

[181] Ben-Dov IZ, Galitzer H, Lavi-Moshayoff V, Goetz R, Kuro-o M, Mohammadi M (2007). The parathyroid is a target organ for FGF23 in rats. J Clin Invest.

[182] Rupp T, Butscheidt S, Vettorazzi E, Oheim R, Barvencik F, Amling M (2019). High FGF23 levels are associated with impaired trabecular bone microarchitecture in patients with osteoporosis. Osteoporos Int.

[183] Ewendt F, Feger M, Föller M (2021). Role of fibroblast growth factor 23 (FGF23) and αKlotho in cancer. Front Cell Dev Biol.

[184] Pazianas M, Miller PD (2021). Osteoporosis and chronic kidney disease-mineral and bone disorder (CKD-MBD): back to basics. Am J Kidney Dis.

[185] Sirikul W, Siri-Angkul N, Chattipakorn N, Chattipakorn SC (2022). Fibroblast growth factor 23 and osteoporosis: evidence from bench to bedside. Int J Mol Sci.

[186] Neves RVP, Corrêa HL, Deus LA, Reis AL, Souza MK, Simões HG (2021). Dynamic not isometric training blunts osteo-renal disease and improves the sclerostin/FGF23/Klotho axis in maintenance hemodialysis patients: a randomized clinical trial. J Appl Physiol (1985).

[187] Cannata-Andía JB, Martín-Carro B, Martín-Vírgala J, Rodríguez-Carrio J, Bande-Fernández JJ, Alonso-Montes C (2021). Chronic kidney disease-mineral and bone disorders: pathogenesis and management. Calcif Tissue Int.

[188] Marzetti E, Calvani R, Tosato M, Cesari M, Di Bari M, Cherubini A (2017). Sarcopenia: an overview. Aging Clin Exp Res.

[189] Mayhew AJ, Amog K, Phillips S, Parise G, McNicholas PD, de Souza RJ (2019). The prevalence of sarcopenia in community-dwelling older adults, an exploration of differences between studies and within definitions: a systematic review and meta-analyses. Age Ageing.

[190] Flanagan-Steet H, Hannon K, McAvoy MJ, Hullinger R, Olwin BB (2000). Loss of FGF receptor 1 signaling reduces skeletal muscle mass and disrupts myofiber organization in the developing limb. Dev Biol.

[191] Pereira SDC, Benoit B, de Aguiar Junior FCA, Chanon S, Vieille-Marchiset A, Pesenti S (2021). Fibroblast growth factor 19 as a countermeasure to muscle and locomotion dysfunctions in experimental cerebral palsy. J Cachexia Sarcopenia Muscle.

[192] Qiu Y, Yu J, Ji X, Yu H, Xue M, Zhang F (2022). Ileal FXR-FGF15/19 signaling activation improves skeletal muscle loss in aged mice. Mech Ageing Dev.

[193] Qiu Y, Yu J, Li Y, Yang F, Yu H, Xue M (2021). Depletion of gut microbiota induces skeletal muscle atrophy by FXR-FGF15/19 signalling. Ann Med.

[194] Roh E, Hwang SY, Yoo HJ, Baik SH, Cho B, Park YS (2021). Association of plasma FGF21 levels with muscle mass and muscle strength in a national multicentre cohort study: Korean Frailty and Aging Cohort Study. Age Ageing.

[195] Minami S, Sakai S, Yamamoto T, Takabatake Y, Namba-Hamano T, Takahashi A (2024). FGF21 and autophagy coordinately counteract kidney disease progression during aging and obesity. Autophagy.

[196] Harrison SA, Rolph T, Knot M, Dubourg J (2024). FGF21 agonists: an emerging therapeutic for metabolic dysfunction-associated steatohepatitis and beyond. J Hepatol.

[197] Kim KH, Jeong YT, Oh H, Kim SH, Cho JM, Kim YN (2013). Autophagy deficiency leads to protection from obesity and insulin resistance by inducing Fgf21 as a mitokine. Nat Med.

[198] Zhang Y, Xie Y, Berglund ED, Coate KC, He TT, Katafuchi T (2012). The starvation hormone, fibroblast growth factor-21, extends lifespan in mice. ELife.

[199] Romanello V (2020). The interplay between mitochondrial morphology and myomitokines in aging sarcopenia. Int J Mol Sci.

[200] Suomalainen A, Elo JM, Pietiläinen KH, Hakonen AH, Sevastianova K, Korpela M (2011). FGF-21 as a biomarker for muscle-manifesting mitochondrial respiratory chain deficiencies: a diagnostic study. Lancet Neurol.

[201] Hanks LJ, Gutiérrez OM, Bamman MM, Ashraf A, McCormick KL, Casazza K (2015). Circulating levels of fibroblast growth factor-21 increase with age independently of body composition indices among healthy individuals. J Clin Transl Endocrinol.

[202] Oost LJ, Kustermann M, Armani A, Blaauw B, Romanello V (2019). Fibroblast growth factor 21 controls mitophagy and muscle mass. J Cachexia Sarcopenia Muscle.

[203] Tezze C, Romanello V, Desbats MA, Fadini GP, Albiero M, Favaro G (2017). Age-associated loss of OPA1 in muscle impacts muscle mass, metabolic homeostasis, systemic inflammation, and epithelial senescence. Cell Metab.

[204] Jin L, Yang R, Geng L, Xu A (2023). Fibroblast growth factor-based pharmacotherapies for the treatment of obesity-related metabolic complications. Annu Rev Pharmacol Toxicol.

[205] Yadav P, Khurana A, Bhatti JS, Weiskirchen R, Navik U (2022). Glucagon-like peptide 1 and fibroblast growth factor-21 in non-alcoholic steatohepatitis: an experimental to clinical perspective. Pharmacol Res.

[206] Lu W, Xiao W, Xie W, Fu X, Pan L, Jin H (2021). The role of osteokines in sarcopenia: therapeutic directions and application prospects. Front Cell Dev Biol.

[207] Kuro-O M (2021). Klotho and calciprotein particles as therapeutic targets against accelerated ageing. Clin Sci (Lond).

[208] Li DJ, Fu H, Zhao T, Ni M, Shen FM (2016). Exercise-stimulated FGF23 promotes exercise performance via controlling the excess reactive oxygen species production and enhancing mitochondrial function in skeletal muscle. Metabolism.

[209] Cai Q, Wu G, Zhu M, Ge HA, Xue C, Zhang QG (2020). FGF6 enhances muscle regeneration after nerve injury by relying on ERK1/2 mechanism. Life Sci.

[210] Huang J, Wang K, Shiflett LA, Brotto L, Bonewald LF, Wacker MJ (2019). Fibroblast growth factor 9 (FGF9) inhibits myogenic differentiation of C2C12 and human muscle cells. Cell Cycle.

[211] Hayashi K, Suzuki A, Terai H, Ahmadi SA, Rahmani MS, Maruf MH (2019). Fibroblast growth factor 9 is upregulated upon intervertebral mechanical stress-induced ligamentum flavum hypertrophy in a rabbit model. Spine.

[212] Habibi H, Suzuki A, Hayashi K, Salimi H, Terai H, Hori Y (2021). Expression and function of FGF9 in the hypertrophied ligamentum flavum of lumbar spinal stenosis patients. Spine J.

[213] Li X (2019). The FGF metabolic axis. Front Med.

[214] Wu P, Shen L, Liu HF, Zou XH, Zhao J, Huang Y (2023). The marriage of immunomodulatory, angiogenic, and osteogenic capabilities in a piezoelectric hydrogel tissue engineering scaffold for military medicine. Mil Med Res.

